# Gender norms and women’s empowerment as barriers to facility birth: A population-based cross-sectional study in 26 Nigerian states using the World Values Survey

**DOI:** 10.1371/journal.pone.0272708

**Published:** 2022-08-18

**Authors:** Helena Litorp, Anna Kågesten, Karin Båge, Olalekan Uthman, Helena Nordenstedt, Mariam Fagbemi, Bi Puranen, Anna-Mia Ekström

**Affiliations:** 1 Department of Global Public Health, Karolinska Institutet, Stockholm, Sweden; 2 Department of Women’s and Children’s Health, Uppsala University, Uppsala, Sweden; 3 Kantar Public, Lagos, Nigeria; 4 World Values Survey, Stockholm, Sweden; 5 Institute for Future Studies, Stockholm, Sweden; Georgia Southern University, UNITED STATES

## Abstract

**Background:**

Central and western Africa struggle with the world’s lowest regional proportion of facility birth at 57%. The aim of the current study was to compare beliefs related to maternal health care services, science/technology, gender norms, and empowerment in states with high vs. low proportions of facility birth in Nigeria.

**Methods:**

Face-to-face interviews were performed as part of a nationally representative survey in Nigeria using a new module to measure values and beliefs related to gender and sexual and reproductive health and rights collected as part the 2018 World Values Survey. We compared beliefs related to maternal health care services, science/technology, gender norms, and empowerment between Nigerian states with facility birth proportions > 50% vs. < 25% as presented in the 2018 Nigerian Demographic Health Survey report. Pearson’s chi-squared test, the independent t-test, and univariable and multivariable logistic and linear regression were used for analyses. Results were also stratified by gender.

**Results:**

Among the 1,273 participants interviewed, 653 resided in states with high and 360 resided in states with low proportions of facility birth. There were no significant differences between the groups in perceived safety of facility birth (96% vs. 94%) and confidence in antenatal care (91% vs 94%). However, in states with low proportions of facility birth, participants had higher confidence in traditional birth attendants (61% vs. 39%, adjusted odds ratio [aOR] 2.1, [1.5–2.8]), men were more often perceived as the ones deciding whether a woman should give birth at a clinic (56% vs. 29%, aOR 2.4 [1.8–3.3]), and participants experienced less freedom over their own lives (56% vs. 72%, aOR 0.56 [0.41–0.76]). Most differences in responses between men and women were not statistically significant.

**Conclusions:**

In order to increase facility births in Nigeria and other similar contexts, transforming gender norms and increasing women’s empowerment is key.

## Introduction

In order to avert unnecessary maternal and perinatal mortality, the World Health Organization recommends all deliveries are attended by a skilled health care professional, and the proportion of births undertaken by a skilled attendant is included as one of two indicators to meet the Sustainable Development Goals (SDG) agenda of reduced maternal deaths by 2030. In line with these targets, the global proportion of births taking place in a health care facility has increased from 62% in 2000–2005 to 81% in 2013–2015 [1]. The proportion of facility birth is, however, unevenly distributed; while many European, American, and Asian countries report facility births near 99%, central and western Africa struggle with the world’s lowest regional proportion of facility birth at 57% [1]. Although upgrading intrapartum care has been stated to be the most effective measure to curb Nigeria’s high maternal mortality ratio of 512 maternal deaths per 100,000 live births (95% confidence interval [CI] 447–578) [2], the national proportion of births that occurred in a health care facility was estimated at only 39% in 2018, with 16% of births in the North-West and 82% of births in the South-East taking place in a health care institution [2]. According to Nigeria Demographic and Health Survey (NDHS) data from 2013, the two most common reasons among Nigerian women for not giving birth in a health care facility are “not necessary” (29%) and “baby born suddenly” (33%) [3].

Several interacting factors at different levels contribute to the continuously low proportion of facility-based delivery in western Africa, including Nigeria. While poor access to health care services historically has been a major barrier to whether or not women give birth in a health care setting [4–6], access, including distance to facilities, is becoming less of a driver as countries expand health care provision in line with the global targets [5, 7–9]. According to the 2013 NDHS, only 14% of respondents in states with facility births < 25% and 34% of respondents in states with facility births > 50% stated costs, distance, or opening hours to be reasons for not giving birth at a facility [3]. Previous literature has also focused on socio-economic factors such as education, wealth, and maternal age as potentially affecting the likelihood of facility birth [4, 7, 8]. A systematic review of 65 studies from sub-Saharan Africa found that maternal education, parity, residence, and relative household wealth were associated with the likelihood of facility birth [6], but as also concluded by Stephenson et al in their study from six African countries, socioeconomic variables seem to have different effects on facility birth in different communities, hence local and cultural influences might be decisive [10]. In Nigeria, disparities in the proportion of facility birth across different geographic regions have failed to be explained by education, wealth, and urban/rural residence [5, 9].

As the ethnographic researcher Kvernflaten argues, women who do not give birth in facilities have their own desires, wishes, and agendas, which are in turn shaped by social relations and gender norms [11]. Increasing attention is now brought to women’s autonomy and such gender norms among family members and peers and their effect on health outcomes [12], including facility birth. For example, a 2013 study from Mali found that preferences and opinions of the mother in law, women’s perception of their self-efficacy, and women’s value in the society were associated with facility birth, but not the husband’s opinion [13]. Other studies conducted in Nigeria and Bangladesh have concluded that women’s role in household decision-making affect the likelihood of giving birth at a health facility [14] and that husband-only decision-making regarding facility birth is associated with lower use of antenatal care services and skilled birth attendance [15]. The effect of women’s decision-making autonomy has also been reported to be more important for women who live in communities that are less supportive of facility birth [16]. Moreover, when considering women’s autonomy, both men’s and women’s view of women’s autonomy need to be considered in order to demonstrate a strong association with the uptake of health care services [17].

In their qualitative study from Cambodia, Matsuoka et al presents a framework of five barriers to facility birth in low- and middle-income countries: financial, physical (including distance to facility), cognitive (including perceptions about quality of health care services and the role of science to improve delivery outcomes), organizational (including availability of services), and social-cultural (including influence from community members and decision-making) [16, 18]. Despite the literature already published, the authors of the aforementioned systematic review from sub-Saharan Africa stress that few studies go beyond demographic and socio-economic variables and that more research is needed to understand regional differences in the proportion of facility birth and whether the association between facility birth and women’s autonomy is independent or biased by other factors [6]. Also, more studies that highlight the role of beliefs and that include all community members, not only the couple, need to be undertaken to understand the context in which maternity health care decisions are made [16]. Drawing on the Matsuoka et al framework, the current study aims to assess differences in beliefs related to 1) maternal health care services and science/technology in general (reflecting cognitive barriers), and 2) gender norms and empowerment (reflecting socio-cultural barriers), in states with high vs. low proportions of facility birth in Nigeria.

## Materials and methods

We conducted a population-based cross-sectional study combining data from the World Values Survey (WVS) and the NDHS, both conducted in Nigeria in 2018. The WVS is a politically and religiously independent multi-disciplinary global research network that has, in partnership with local organizations, collected data on values, attitudes, and beliefs since 1981. The WVS database covers over 100 countries that are home to over 90% of the world’s population, funded by multiple private and governmental sources. The data are freely available online with approximately 28 billion downloads since 1981. Every five years, a team lead by social scientists collects a new wave of data in each country. The standard WVS includes 291 items and covers beliefs and norms related to homosexuality, sex work, abortion, premarital sex, casual sex, gender-based violence and divorce, the role of women in society, and topics such as subjective health status, happiness, empowerment, and life satisfaction. It also provides a number of indexes to measure constructs such as choice, voice, autonomy, and equality [19], which have previously been demonstrated high correlation with sexual freedom [20], as well as an emancipative worldview in a broader sense [20, 21].

In 2018, the WVS global secretariat in collaboration with the Global and Sexual Health research group at Karolinska Institutet designed a new set of questions to assess gender-related norms and values about sexual and reproductive health and rights (SRHR). The new questions on norms and values related to gender and SRHR were developed in several steps. We first reviewed all SDGs, in particular SDGs 3 on health and well-being for all and SDG 5 on gender equality, to align the questions to the SRHR issues of highest priority to meet the 2030 SDG agenda. Next, we reviewed the standard WVS questionnaire to identify gaps related to gender norms and SRHR. When developing the new items, we reviewed questions that had been validated in other surveys [22–24]. We also constructed questions related to gender in relation to decision making, as such questions were lacking in the standard WVS. When finalized, the new questions were added to the standard WVS questionnaire.

### Study setting

Nigeria is a lower-middle-income country and the 7th most populous country in the world with 188 million inhabitants. Administratively, Nigeria is divided into six geopolitical regions (North-Central, North-East, North-West, South-East, South-South, and South-West) and 36 states. Except for the large regional disparities in the proportion of institutional deliveries as stated above, there are extensive geographical differences particularly between the southern and northern parts of the country with northern states having lower levels of wealth, school attendance, and women’s ownership of assets, bank accounts, and mobile phones as well as role in decision making [2].

### Data collection and population

The new gender and SRHR questions were added to the standard WVS items as part of the 7th WVS wave conducted in Nigeria between December 2017 and January 2018. Nigeria was selected as pilot setting due to its high burden of adverse SRHR outcomes. In addition, Nigeria is home to a range of different ethnic groups, languages and religions, thus provides optimal conditions to evaluate the module within and across different socio-cultural contexts. An initial version of the translated questionnaires was pre-piloted in the four largest local languages (hausa, igbo, yoruba,and fulfulde) to assess the understanding and interpretation of the questions across different communities, followed by revisions into a final version ready for the full survey. The interview guide for data collection for the WVS can be found in S1 Appendix.

The target population was men and women aged over 18 years. The sampling procedure followed the standard WVS rules of conduct for highest possible internal and external validity: 1) selection of all geopolitical regions using population proportionate to size; 2) selection of states in each region using population proportionate to size; and 3) selection of local government areas considering the minimum number of interviews needed per unit [25]. Data collection was conducted by a local research organization, trading as Kantar Public. The field team was led by the same scientist as in the two latest WVS surveys in Nigeria, and consisted of 42 trained interviewers, 21 supervisors, and 11 quality control officers. Face-to-face, household interviews were conducted with the household members who were at home at the time of the interview using Samsung tablets as data collection devices. All of the interviews were also audio-recorded and listened to by in-house quality personnel. To ensure data quality, supervisor sit-ins, monitoring by quality control officers, audio-recording, and telephone back-checking were used. In total, 20% of the interviews were back-checked by a supervisor, 5% were accompanied by a supervisor, and 15% were back-checked by an independent quality control officer.

To estimate the proportions of facility birth, we used data as presented in the 2018 NDHS report [2]. The 2018 NDHS was the sixth survey of its kind to be conducted in Nigeria since 1990, with the objective to provide reliable estimates on a range of health outcomes and determinants. In the NDHS, a representative sample of approximately 42,000 households was selected and proportions of facility birth were calculated among all women aged 15–49 who had had a birth within the five proceeding years (N = 12,935) who were either permanent residents of the selected households or visitors who stayed in the household the night before the survey.

### Independent variable: High vs. low proportions of facility birth

For the purpose of the current study, and in the absence of individual data on facility birth in the WVS, we constructed a proxy measure to reflect high vs. low proportions of facility birth according to data from the 2018 NDHS report [2]. Specifically, we grouped states with high proportions of facility birth, arbitrary defined as > 50% according to the NDHS report, and states with low proportions of facility birth, defined as < 25%. Based on the grouping, each participant in the WVS database was assigned the corresponding code (high vs. low proportion of facility birth) based on the respondent’s state of inclusion. As the purpose of the current study was to highlight differences between states with high vs. low proportions of facility birth, participants who lived in states with proportions of facility birth between 25% and 50% were excluded from the analyses. The method of analyzing WVS data in relation to outcomes registered in other sources has been used previously [20, 21], for example by Alexander et al who compared emancipative values on sexual freedom in relation to average life expectancy, fertility rates, and per capita gross domestic product to showcase the association between these variables [20].

### Dependent variables: Beliefs related to maternal health care services, science/technology in general, and gender norms/empowerment

Drawing on the framework suggested by Matsuoka et al [18], we focused on beliefs related to maternal health care services, science/technology in general, and beliefs related to gender norms and empowerment as potential barriers to institutional delivery. Specifically, we hypothesized that participants who lived in states with higher proportions of facility birth would express more positive perceptions of maternal health care services and of science and technology in general. Given the role of gender equality and women’s agency in shaping decisions about where to give birth [13–15], we further hypothesized that beliefs related to gender norms and empowerment would be more traditional or stereotypical in states with low proportions of facility birth.

To assess beliefs related to the quality of maternal health care services, we included 10 statements asking participants about their confidence in different health care services and their attitudes towards science and technology in general, given the role of science/technology in institutional delivery care; 1) “It is safer for a woman to give birth in a facility than at home”, 2–8) “I have confidence in…” (hospitals, doctors, midwives at the clinic, safe delivery at the health facility, antenatal services, traditional birth attendants, respectively), 9) “Science and technology are making our lives easier and more comfortable”, and 10)”Whenever science and religion conflict, religion is always right”. All items were measured on a scale from 1 (“strongly agree”) to 4 (“strongly disagree”), except item 9 (science/technology) which used a 10-point scale (1 =“strongly disagree”, 10 = “strongly agree”). All scales were dichotomized into high (1–2 for the 4-grade scale, 1–5 for the 10-grade scale) versus low agreement (3–4 vs. 6–10, respectively).

To assess beliefs related to gender norms and empowerment, we included the following questions: “In the community where you live, who usually decides over health care visits and spending/if a woman should give birth in a clinic/over major household purchases?”. The variable on decisions regarding household purchases was chosen as there were previous reports of it being associated with facility birth [15]. Variables on decision-making were given on a 9 grade scale, where 1 represented men-only decision making, 5 represented equal decision making, and 9 represented women-only decision making. For the purpose of this study, we considered 1–4 as “men decide”. We regarded the questions on decision-making to be descriptive social norms, as they reflected the participants’ perception of what was commonly done in a specific situation in their respective communities. Given the previous discussion on the role of autonomy in relation to facility birth [4, 6, 10, 13–16], we also included the variable “I experience freedom over my own life”, and the WVS choice, voice, autonomy, and equality indexes [19] as representing beliefs related to gender norms and empowerment [20, 21]. The choice index was coded by WVS by combining norms related to homosexuality, abortion, and divorce, the voice index was coded by WVS based on overall emancipative values, the autonomy index was coded by WVS by combining views on independence, imagination, and nonobedience, and the equality index was coded by WVS by combining norms on women’s education, women’s employment, and women as politicians [19]. All four indexes were standardized on a 0–1 scale, with higher scores representing greater support for each construct [20, 21].

### Analysis

Data were analyzed using Statistical Package for the Social Sciences (SPSS) version 25 [26]. We compared background characteristics of participants in states with high vs. low proportions of facility birth by descriptive statistics and analyzed beliefs by using proportions of participants agreeing vs. disagreeing to the statements. Proportions were compared between the two groups using the Pearson chi-square test. For all variables and for all WVS indexes, we also calculated the mean values, including standard deviations, and compared means between the groups using the independent t-test. *p*-values < 0.05 were considered statistically significant.

To further analyze differences between the groups, we calculated the crude and adjusted odds ratios (cOR and aOR), including 95% CIs, using univariable and multivariable logistic regression. Mean values for WVS indexes were analyzed using multivariable linear regression. For the multivariable analysis, we included education, religion, place of residence (urban/per-urban/rural), and subjective social class (indicated by the participants as lower/working/lower-middle/upper-middle or upper class) as co-variates, given that previous literature indicated these factors to be potential confounders [4, 6–8]. We also performed a sub-analysis stratifying all results for gender as well as compared beliefs in states with high vs. low proportions of facility birth only in the north to explore generalizability of the findings. The WVS dataset is available both weighted and unweighted, but for the current study unweighted data was used as the sample was considered nationally representative. The WVS wave 7 dataset will be publicly available on the WVS web site during 2021 [25].

### Ethical considerations

The study procedures followed the Code of Professional Ethics and Practices drawn by the WVS and was approved by the Swedish Ethical Review Authority (reference number 2020–05314). The data set used by the researchers contained no identifying information regarding participants, whose anonymity was thereby fully ensured. Verbal informed consent was sought from all respondents prior to their participation and was chosen for practical reasons instead of written informed consent. The verbal informed consent contained all elements of a written informed consent and using verbal consent did not adversely affect the rights or wellbeing of the subjects as compared to using written informed consent.

## Results

The seventh wave of the WVS in Nigeria included 1,237 participants aged 18 to 100 years recruited from all major states in Nigeria. The new gender and SRHR module was found to be highly acceptable and easy to understand for both men and women across all age groups. For the current study, we included 1,013 participants, of which 653 were recruited from states with high proportions of facility birth and 360 were recruited from states with low proportions of facility birth (Table 1). We excluded 224 participants who lived in states with proportions of facility birth between 25% and 50%. Missing values comprised 0–6% of responses for the respective variables and were excluded from the analyses of that variable.

**Table 1:** Proportion of institutional deliveries in states with high vs. low proportions of facility birth in Nigeria.

State	Geopolitical region	Proportion of facility birth in 2018 Nigeria Demographic and Health Survey	Number of participants in 2018 World Values Survey
**States with high proportions of facility birth**			
Imo	South-East	95%	30
Abia	South-East	92%	19
Osun	South-West	92%	41
Anambra	South-East	90%	40
Ondo	South-West	81%	32
Edo	South-South	80%	40
Enugu	South-South	80%	30
Lagos	South-West	76%	90
Ogun	South-West	73%	50
Kogi	North-Central	72%	44
Ekiti	South-West	72%	33
Oyo	South-West	70%	60
Benue	North-Central	67%	33
FCT-Abuja	North-Central	63%	30
Delta	South-South	55%	30
Kwara	North-Central	55%	21
Cross River	South-South	53%	30
*Total*		*74%*	*653*
**States with low proportions of facility birth**			
Bayelsa	South-South	23%	10
Bauchi	North-East	22%	41
Jigawa	North-West	20%	40
Kano	North-West	19%	71
Kaduna	North-West	18%	55
Katsina	North-West	17%	50
Yobe	North-East	16%	11
Zamfara	North-West	11%	20
Kebbi	North-West	7.4%	20
Sokoto	North-West	7.8%	42
*Total*		*16%*	*360*

Characteristics of study participants are illustrated in Table 2. There were no differences in age distribution or gender, but participants in states with low proportions of facility birth more often resided in rural areas, had lower educational level, and lower subjective social class. A larger proportion of participants in states with high proportions of facility birth were also single and denominated themselves as Christians, while the majority in states with low proportions of facility birth were Muslims.

**Table 2:** Characteristics of participants in the 2018 Nigeria World Values Survey.

Characteristic	States with high proportions of facility birth N (%)	States with low proportions of facility birth N (%)	Total N (%)	*p-*value
**Age**				
18–25	219 (34%)	136 (38%)	355 (35%)	
26–35	213 (33%)	119 (33%)	332 (33%)	
36–50	156 (24%)	76 (21%)	232 (23%)	
> 50	65 (10%)	27 (8.4%)	94 (9.3%)	0.41
**Gender**				
Women	322 (49%)	175 (49%)	497 (49%)	
Men	331 (51%)	185 (51%)	516 (51%)	0.83
**Residence**				
Urban	419 (64%)	114 (32%)	533 (53%)	
Peri-urban	113 (17%)	114 (32%)	227 (22%)	
Rural	121 (19%)	132 (37%)	253 (25%)	< 0.001
**Education**				
Early childhood/No education	122 (19%)	60 (17%)	182 (18%)	
Primary education	76 (12%)	49 (14%)	125 (12%)	
Lower secondary education	43 (6.6%)	89 (25%)	132 (13%)	
Upper secondary education	280 (43%)	105 (29%)	385 (38%)	
Post-secondary non-tertiary education	54 (8.3%)	35 (9.7%)	89 (8.8%)	
University	69 (11%)	22 (6.1%)	91 (9.0%)	< 0.001
**Subjective social class** ^a^				
Upper class	20 (3.1%)	10 (2.8%)	30 (3.0%)	
Upper middle class	73 (11%)	39 (11%)	112 (11%)	
Lower middle class	197 (30%)	80 (22%)	277 (27%)	
Working class	138 (21%)	28 (7.8%)	166 (16%)	
Lower class	213 (33%)	194 (54%)	407 (40%)	< 0.001
**Marital status**				
Married/co-habiting	344 (53%)	231 (64%)	575 (57%)	
Single	276 (42%)	113 (31%)	389 (38%)	
Widowed/Divorced/Separated	31 (4.8%)	15 (4.3%)	46 (4.6%)	0.004
**Religion**				
Muslim	169 (26%)	268 (74%)	437 (43%)	
Protestant	300 (46%)	33 (9.0%)	333 (33%)	
Catholic	129 (20%)	17 (4.7%)	146 (14%)	
Orthodox	21 (3.2%)	4 (1.1%)	25 (2.5%)	
Other/Do not belong to a religious denomination	24 (4.3%)	36 (10%)	60 (6.3%)	< 0.001
**Total**	653 (100%)	360 (100%)	1,013 (100%)	

^a^ As indicated by the participants.

As presented in Table 3, the majority of participants in states with both high and low proportions of facility birth agreed that it was safer for a woman to give birth in a facility than at home (96% and 94% respectively) and reported high confidence in antenatal care (91% vs 94%). Participants in states with low proportions of facility birth, however, more often had confidence in hospitals (86% vs. 69%, *p* < 0.001), doctors (1.4 vs. 1.5, *p* = 0.004), midwives at the clinic (85% vs 79%, *p* = 0.02), and traditional birth attendants (61% vs. 39%, *p* < 0.001) than participants in states with high proportions of facility birth. On the other hand, participants in states with high proportions of facility birth had higher trust in science and technology.

**Table 3:** Beliefs related to maternal health care services and science/technology in Nigeria.

Beliefs related to maternal health care services and science/technology		States with high proportions of facility birth	States with low proportions of facility birth	*p*-value ^a^
It is safer for a woman to give birth at a clinic than at home	N (%)	622 (96%)	337 (94%)	0.15
	Mean value 1–4^b^ (SD)	1.4 (0.61)	1.4 (0.68)	0.34
I have confidence in hospitals	N (%)	451 (69%)	308 (86%)	< 0.001
	Mean value 1–4^c^ (SD)	2.1 (0.94)	1.6 (0.79)	< 0.001
I have confidence in doctors	N (%)	590 (92%)	336 (94%)	0.22
	Mean value 1–4^c^ (SD)	1.5 (0.69)	1.4 (0.61)	0.004
I have confidence in midwives at the clinic	N (%)	501 (79%)	305 (85%)	0.02
	Mean value 1–4^c^ (SD)	1.9 (0.85)	1.6 (0.79)	< 0.001
I have confidence that health care facilities can provide safe delivery services	N (%)	574 (90%)	333 (93%)	0.12
	Mean value 1–4^c^ (SD)	1.5 (0.89)	1.4 (0.73)	0.04
I have confidence in antenatal care	N (%)	574 (91%)	335 (94%)	0.15
	Mean value 1–4^c^ (SD)	1.4 (0.92)	1.3 (0.71)	0.19
I have confidence in traditional birth attendants	N (%)	240 (39%)	217 (61%)	< 0.001
	Mean value 1–4 ^d^ (SD)	2.8 (0.99)	2.3 (1.1)	< 0.001
Science and technology are making our lives healthier, easier, and more comfortable	N (%)	569 (88%)	289 (80%)	0.002
	Mean value 1–10^d^ (SD)	8.3 (2.4)	7.8 (2.4)	0.002
Whenever science and religion conflict, religion is always right	N (%)	559 (89%)	342 (95%)	< 0.001
	Mean value 1–4^b^ (SD)	1.6 (0.87)	1.3 (0.61)	< 0.001
**Total**		653	360	

^a^ Pearson chi-square for proportions and independent t-test for continuous variables

^b^ Strongly agree = 1, Strongly disagree = 4

^c^ A great deal of trust = 1, No trust at all = 4

^d^ Completely disagree = 1, Completely agree = 10

Statements reflecting beliefs related to gender norms and empowerment revealed large discrepancies between states with high vs. low proportions of facility birth (Table 4). In states with low proportions of facility birth, men were more often perceived to make decisions over health care visits and spending (59% vs. 36%, *p* < 0.001) and whether a woman should give birth at a clinic (56% vs. 29%, *p* < 0.001). Men and women in states with low proportions of facility birth also perceived they had less agency over their own lives (56% vs. 72%, *p* < 0.001). Furthermore, support for all WVS sub-indexes related to choice, voice, autonomy, and gender equality was lower in states with low as compared to high proportions of facility birth, indicating lower support for individual freedom and agency in general, and women’s empowerment particularly.

**Table 4:** Beliefs related to gender norms and empowerment in Nigeria.

Beliefs related to gender norms and empowerment		States with high proportions of facility birth	States with low proportions of facility birth	*p*-value ^a^
In the community where I live, men usually decide over health care visits and spending	Men decide N (%)	231 (36%)	214 (59%)	< 0.001
	Mean value 1–9^b^ (SD)	4.1 (2.3)	3.0 (2.2)	< 0.001
In the community where I live, men usually decide if a woman should give birth at a clinic	Men decide N (%)	190 (29%)	200 (56%)	< 0.001
	Mean value 1–9^b^ (SD)	4.6 (2.4)	3.2 (2.4)	< 0.001
In the community where I live, men usually decide over major household purchases	Men decide N (%)	227 (35%)	239 (66%)	< 0.001
	Mean value 1–9^b^ (SD)	4.4 (2.6)	2.7 (2.3)	< 0.001
I experience freedom over my own life	N (%)	470 (72%)	202 (56%)	< 0.001
	Mean value 1–10^c^ (SD)	7.2 (2.7)	6.0 (2.9)	< 0.001
Choice sub-index^d^	Mean value 0–1^d^ (SD)	0.12 (0.17)	0.08 (0.13)	< 0.001
Voice sub-index^d^	Mean value 0–1^d^ (SD)	0.37 (0.26)	0.34 (0.25)	0.03
Autonomy sub-index ^d^	Mean value 0–1^d^ (SD)	0.33 (0.31)	0.25 (0.27)	< 0.001
Gender equality index^d^	Mean value 0–1^d^ (SD)	0.43 (0.27)	0.24 (0.24)	< 0.001
**Total**		653	360	

^a^ Pearson chi-square for proportions and independent t-test for continuous variables

^b^ Men decide = 1, Both decide = 5, Women decide = 9

^c^ No choice at all = 1, A great deal of choice = 10

^d^ Higher value indicates more choice (coded by combining norms related to homosexuality, abortion, and divorce), voice (coded based on overall emancipative index), autonomy (coded by combining perceptions on independence, imagination, and nonobedience), and equality (coded by combining norms on women’s education, women’s employment, and women as politicians).

Crude and adjusted ORs from the multivariable analyses are presented in Table 5. Differences in statements reflecting gender norms and empowerment between states with high vs. low proportions of facility birth remained statistically significant after adjusting for education, religion, residence, and subjective social class, while the difference in trust in midwives failed to reach statistical significance after adjustment. Participants in states with low proportions of facility birth had higher confidence in hospitals (aOR 2.3, 95% CI 1.6–3.4), traditional birth attendants (aOR 2.1, 95% CI 1.5–2.8), and religion as compared to science (aOR 2.3, 95% CI 1.3–4.3). In such states, men were also more often perceived as the ones making decisions over health care visits and spending (aOR 2.2, 95% CI 1.6–2.9) and whether a woman should give birth at a clinic (aOR 2.4, 95% CI 1.8–3.3), and participants experienced less freedom over their own lives (aOR 0.56, 95% CI 0.41–0.76). In the multivariable linear regression of WVS indexes, all *p*-values remained statistically significant after adjusting for education, religion, residence, and subjective social class.

**Table 5:** Multivariable logistic regression of likelihood of participants in states with low proportions of facility birth agreeing to the statements.

	cOR (95% CI)	aOR (95% CI) adjusted for education^a^	aOR (95% CI) adjusted for religion^b^	aOR (95% CI) adjusted for residence^c^	aOR (95%CI) adjusted for subjective social class^d^	aOR (95% CI) adjusted for education, religion, residence and subjective social class^a^^,^ ^b^^,^ ^c^^,^ ^d^
**Beliefs related to maternal health care services and science/technology**						
I have confidence in hospitals	2.6 (1.9–3.7)	2.6 (1.8–3.6)	2.2 (1.5–3.1)	2.9 (2.1–4.1)	2.6 (1.8–3.6)	2.3 (1.6–3.4)*
I have confidence in midwives at the clinic	1.5 (1.1–2.2)	1.5 (1.1–2.2)	1.5 (1.0–2.1)	1.6 (1.1–2.3)	1.5 (1.1–2.2)	1.5 (1.0–2.2)
I have trust in traditional birth attendants	2.5 (1.9–3.2)	2.4 (1.9–3.2)	2.2 (1.7–2.3)	2.3 (1.8–3.1)	2.4 (1.9–3.2)	2.1 (1.5–2.8)*
Science and technology are making our lives healthier, easier, and more comfortable	0.58 (0.41–0.82)	0.58 (0.41–0.83)	0.59 (0.41-.0.86)	0.56 (0.39–0.81)	0.60 (0.42–0.85)	0.58 (0.39–0.86)*
Whenever science and religion conflict, religion is always right	2.6 (1.5–4.5)	2.5 (1.5–4.4)	2.2 (1.3–3.8)	2.9 (1.6–5.1)	2.6 (1.5–4.4)	2.3 (1.3–4.3)*
**Beliefs related to gender norms and empowerment**						
In my community, men usually decide over health care visits and spending	2.6 (2.0–3.5)	2.6 (2.0–3.4)	2.2 (1.6–2.9)	2.6 (2.0–3.5)	2.6 (2.0–3.4)	2.2 (1.6–2.9)*
In my community, men decide if a woman should give birth at a clinic	3.0 (2.3–3.9)	3.0 (2.3–3.9)	2.5 (1.9–3.3)	2.9 (2.2–3.9)	3.0 (2.3–3.9)	2.4 (1.8–3.3)*
I experience freedom over my own life	0.49 (0.38–0.65)	0.50 (0.38–0.66)	0.52 (0.39–0.70)	0.52 (0.39–0.69)	0.51 (0.439–0.66)	0.56 (0.41–0.76)*

^a^ No education, primary school, lower secondary, upper secondary, postsecondary nontertiary, and university

^b^ Muslim, Protestant, Christian, Orthodox, and other/do not belong to a religious denomination

^c^ Urban, peri-urban, and rural

^d^ Upper class, upper middle class, lower middle class, working class, and lower class, as indicated by the respondents

*Significant associations in the final model

When stratifying the results based on gender, there were no differences in responses between men and women in beliefs related to trust in maternal health care services and science/technology (Table A in S2 Appendix). However, in states with low proportions of facility birth, men more often experienced freedom over their own lives than women (62% vs 50%, *p* = 0.02), while no such difference was observed between men and women in states with high proportions of facility birth (Table B in S2 Appendix). Men and women in both groups agreed on who usually decided if a woman should give birth at a health care facility, but men and women in states with high proportions of facility birth disagreed on who usually made decisions regarding health care visits and spending: 31% of women vs. 41% of men declared men to make these decisions (*p* = 0.007), while no such disagreement was detected between men and women in states with low proportions of facility birth (Table B in S2 Appendix). There were no differences between men and women with regards to the WVS indexes analyzed except for the gender equality index, with women being more supportive to gender equality than men in states with high as well as low proportions of facility birth (Table B in S2 Appendix). To account for the potential bias that most states with high proportions of facility birth were in the south Nigeria and most states with low proportions of facility birth were in the north, we also performed a sub-analysis comparing states with high vs. low proportions of facility birth only in the north (Tables C, D in S2 Appendix). Results for the northern states corresponded to the results of the total study population except for the variable freedom over one’s own life, for which there were no statistically significant differences between northern states with high vs. low proportions of facility birth.

## Discussion

While exploring Nigeria’s huge geographical discrepancies with regards to the proportion of facility birth, we found that states with low proportions of facility birth were not characterized by lower perceived safety of facility birth or low confidence in health care professionals or institutions, but rather that in such states men were more often perceived to make decisions regarding facility birth and health care visits and respondents, especially women, experienced less freedom over their lives than participants in states with high proportions of facility birth. Despite the fact that states with low proportions of facility birth were characterized by lower education level, a higher percentage of respondents denominating themselves as Muslim, a higher proportion of rural residents, and lower subjective social class, all differences in gender norms and empowerment between states with high vs. low proportions of facility birth remained statistically significant after adjusting for education, religion, residence, and subjective social class.

In contrast to how previous studies have found that fear of poor quality of care [13, 27, 28], low trust in local doctors [29], and perceived equivalent skills between traditional birth attendants and health care professionals [6] affect the likelihood of facility birth, participants in our study, both residing in states with high and low proportions of facility birth, reported high confidence in facility birth, health care professionals, and health care institutions. Our study can only speculate on reasons for the paradoxically high trust in maternal health care institutions yet low proportions of facility birth, hence further exploring these findings using qualitative methods might provide a deeper understanding to guide policy and implementation [12]. As described before [6, 28, 29], higher trust in traditional birth attendants seemed to be associated with low proportions of facility birth, emphasizing the importance of addressing and potentially involving this cadre in order to increase facility births.

In line with previous studies from Nigeria and other low- and middle-income countries [4–6, 10, 13–16], we found male decision-making to be statistically significant and consistently associated with residing in a state with low proportion of facility birth, even after adjusting for differences between the states in education level, religion, residence, and subjective social class. We also found that participants from states with low proportions of facility birth were less likely than those from states with high proportions of facility birth to report freedom and control over their own lives, and participants in states with low proportions of facility birth demonstrated lower support for different components of empowerment such as voice, choice, autonomy, and gender equality. As emphasized by the “belief-mediation” theory, social practices persist because they are inspired by subjective beliefs about what is legitimate and desirable [21]. Hence, women’s empowerment, for example regarding decisions on their use of health care services, will be dependent on subjective beliefs in the society about the legitimacy and desirability of female decision-making [21]. Our study stresses the importance of integrating gender norms into maternal and child health policies [12], as currently done in The Nigeria Maternal and Child Survival Program [30].

This is the first study presenting results from a newly developed module in the WVS specifically focusing on gender and SRHR. The main strengths of the study include the population-based sample and thorough validity checks, which are both characteristics of the WVS data collection [25]. Consequently, missing data was kept to a minimum. Another strength is the global distribution of the WVS, which will allow for comparing our results with findings from other countries and regions, given that the gender and SRHR module is to be included in future WVS waves. A potential bias with our study is that, like most other surveys on gender norms [12], the WVS did not measure health outcomes such as facility birth among the individual participants. Instead, we used the proportions of facility birth as reported in the NDHS of the state in which the participant was included as a proxy. This method, i.e. analyzing WVS data on values in relation to health outcomes registered in other data sources, has been employed successfully in previous studies [20, 21]. Given the design, it is possible that ecological fallacy affected our results; however, as the goal was to compare differences in beliefs at the community level (high vs. low proportion states), we still believe that the combination of NDHS and WVS data fulfilled this purpose. There were also significant differences between the two groups of comparison in terms of demographic and socio-economic characteristics, which would most likely have affected the results, but the main results remained statistically significant after adjusting for these factors. The sub-analysis we conducted to compare states with high vs. low proportions of facility birth only in the north yielded similar findings as the ones presented in this study, which strengthens the generalizability of our findings despite the geographical aggregation of states with high proportions of facility birth in the south.

## Conclusion

We found that states with low proportions of facility birth were characterized by men being perceived as the main decision-makers, less support for science/technology, gender equality and empowerment, and higher confidence in traditional birth attendants. On the contrary, we found similar or even higher trust in perceived safety of facility birth and health care professionals and institutions in states with low proportions of facility birth compared to states with high proportions of facility birth. Our results indicate that interventions aiming to increase facility births need to address social norms and beliefs related to gender and empowerment, including women’s agency and decision-making. Such interventions need to target both men and women, but particularly men as women were generally more supportive of norms and values related to gender equality. Given the population-based sample, we believe our results to be generalizable to other low- and middle-income countries, and hope they may help policy-makers design and tailor interventions to increase facility births in order to reach the SDG 2030 targets. Including the newly developed gender and SRHR module into future WVS waves, especially in countries with poor reproductive health outcomes, would allow for cross-national, longitudinal analyses of beliefs and values in relation to reproductive health on a global level and further improve the possibility to design effective policies.

## Supporting information

S1 Appendix Interview guide of the Nigeria World Values Survey wave 7. (PDF)

S2 Appendix Sub-analyses by gender and northern states. (DOCX)
