# Supplementary material for: Gender norms and women’s empowerment as barriers to facility birth: A population-based cross-sectional study in 26 Nigerian states using the World Values Survey
Source: PLoS One. 2022 Aug 18;17(8):e0272708. doi: 10.1371/journal.pone.0272708 (PMC9387800; doi:10.1371/journal.pone.0272708)
Supplement: S1 Appendix — (PDF) [file pone.0272708.s001.pdf]

2017 -2018 WORLD VALUES SURVEY WAVE 7

including module on gender and family planning

Pages: 25-30

December 13th, 2017

For comments please email

bi.puranen@worldvaluessurvey.org

Coding for missing codes is as follows (do not read them and code only if the respondent mentions them him-/herself):<br>-1 Don't know -3 Not applicable (filter)<br>-2 No answer/refused -5 Missing; Not applicable for other reasons

INTRODUCTION: Hello Sir/Ma. I am from the TNS Nigeria Limited. We are carrying out a global study of what people value in life. This study will interview samples representing most of the world's people. Your name has been selected at random as part of a representative sample of the people in Nigeria. I'd like to ask your views on a number of different subjects. Your input will be treated strictly confidential, but it will contribute to a better understanding of what people all over the world believe and want out of life. Thank you very much for your collaboration. Can I proceed?

Yes…………………………………………1
No…………………………………………..2

**A.**	**SURVEY WAVE NUMBER**	7

**B.**	**COUNTRY CODE**	5	6	6

**C.**	**COW COUNTRY CODE**	4	7	5

**D.**	**INTERVIEW NUMBER** *(4-digit questionnaire number)*				

D1. REGION

South West	1
South South	2
South East	3
North West	4
North East	5
North Central	6
Lagos	7

**D2.**	**STATE CODE**			**D3.**	**ISO STATE CODE**	

State	D2	D3		State	D2	D3
Lagos	1	NG-LA		Kwara	20	NG-KW
Oyo	2	NG-OY		Niger	21	NG-NI
Edo	3	NG-ED		Benue	22	NG-BE
Akwa Ibom	4	NG-AK		Nasarawa	23	NG-NA
Enugu	5	NG-EN		Delta	24	NG-DE
Abia	6	NG-AB		Bayelsa	25	NG-BY
Kano	7	NG-KN		Cross River	26	NG-CR
Kaduna	8	NG-KD		Anambra	27	NG-AN
Borno	9	NG-BO		Ebonyi	28	NG-EB
Bauchi	10	NG-BA		Imo	29	NG-IM
Plateau	11	NG-PL		Gombe	30	NG-GO
Abuja	12	NG-FC		Yobe	31	NG-YO
Rivers	13	NG-RI		Taraba	32	NG-TA
Sokoto	14	NG-SO		Jigawa	33	NG-JI
Adamawa	15	NG-AD		Zamfara	34	NG-ZA
Ogun	16	NG-OG		Kebbi	35	NG-KE
Ondo	17	NG-ON		Katsina	36	NG-KT
Ekiti	18	NG-EK		Kogi	37	NG-KO
Osun	19	NG-OS				

I. Code Primary Sampling Unit number

J. Date of the interview:

Date		Month		Year			

K1. Time of the interview:

Start hour		Start minute	

Coding for missing codes is as follows (do not read them and code only if the respondent mentions them him-/herself):
-1 Don't know -3 Not applicable (filter)
-2 No answer/refused -5 Missing; Not applicable for other reasons

SOCIAL VALUES, ATTITUDES & STEREOTYPES

(***SHOW CARD 1***)
For each of the following, indicate how important it is in your life. Would you say it is (*read out and code one answer for each*):

		Very important	Rather important	Not very important	Not at all important
Q1	Family	1	2	3	4
Q2	Friends	1	2	3	4
Q3	Leisure time	1	2	3	4
Q4	Politics	1	2	3	4
Q5	Work	1	2	3	4
Q6	Religion	1	2	3	4

(***SHOW CARD 2***)
Here is a list of qualities that children can be encouraged to learn at home. Which, if any, do you consider to be especially important? Please choose up to five! (*Code five mentions at the maximum*):

		Mentioned	Not mentioned
Q7	Good manners		
Q8	Independence	1	2
Q9	Hard work	1	2
Q10	Feeling of responsibility	1	2
Q11	Imagination	1	2
Q12	Tolerance and respect for other people	1	2
Q13	Thrift, saving money and things	1	2
Q14	Determination, perseverance	1	2
Q15	Religious faith	1	2
Q16	Not being selfish (unselfishness)	1	2
Q17	Obedience	1	2
		1	2

(***SHOW CARD 3***)
On this list are various groups of people. Could you please mention any that you would not like to have as neighbors? (*Code an answer for each group*):

		Mentioned	Not mentioned
Q18	Drug addicts	1	2
Q19	People of a different race	1	2
Q20	People who have AIDS	1	2
Q21	Immigrants/foreign workers	1	2
Q22	Homosexuals	1	2
Q23	People of a different religion	1	2
Q24	Heavy drinkers	1	2
Q25	Unmarried couples living together	1	2
Q26	People who speak a different language	1	2

Coding for missing codes is as follows (do not read them and code only if the respondent mentions them him-/herself):
-1 Don't know -3 Not applicable (filter)
-2 No answer/refused -5 Missing; Not applicable for other reasons

For each of the following statements I read out, can you tell me how strongly you agree or disagree with each. Do you strongly agree, agree, disagree, or strongly disagree? (*Read out and code one answer for each statement*):

		Strongly agree	Agree	Disagree	Strongly disagree
Q27	One of my main goals in life has been to make my parents proud	1	2	3	4
Q28	When a mother works for pay, the children suffer	1	2	3	4
Q29	On the whole, men make better political leaders than women do	1	2	3	4
Q30	A university education is more important for a boy than for a girl	1	2	3	4
Q31	On the whole, men make better business executives than women do	1	2	3	4
Q32	Being a housewife is just as fulfilling as working for pay	1	2	3	4

How would you feel about the following statements? Do you agree or disagree with them?

		Agree strongly	Agree	Neither agree nor disagree	Disagree	Disagree strongly
Q33	When jobs are scarce, men should have more right to a job than women	1	2	3	4	5
Q34	When jobs are scarce, employers should give priority to people of this country over immigrants	1	2	3	4	5
Q35	If a woman earns more money than her husband, it's almost certain to cause problems	1	2	3	4	5
Q36	Homosexual couples are as good parents as other couples	1	2	3	4	5
Q37	It is a duty towards society to have children	1	2	3	4	5
Q38	Adult children have the duty to provide long-term care for their parents	1	2	3	4	5
Q39	People who don't work turn lazy	1	2	3	4	5
Q40	Work is a duty towards society	1	2	3	4	5
Q41	Work should always come first, even if it means less spare time	1	2	3	4	5

(*SHOW CARD 4*)

Q42. **On this card are three basic kinds of attitudes concerning the society we live in. Please choose the one which best describes your own opinion?** *(Please, code only one option from the list below)*

1 The entire way our society is organized must be radically changed by revolutionary action
2 Our society must be gradually improved by reforms
3 Our present society must be valiantly defended against all subversive forces

I'm going to read out a list of various changes in our way of life that might take place in the near future. Please tell me for each one, if it were to happen, whether you think it would be a good thing, a bad thing, or don't you mind? (*Code one answer for each*):

		Good	Don't mind	Bad
Q43	Less importance placed on work in our lives	1	2	3
Q44	More emphasis on the development of technology	1	2	3
Q45	Greater respect for authority	1	2	3

Coding for missing codes is as follows (do not read them and code only if the respondent mentions them him-/herself):	
-1 Don't know	-3 Not applicable (filter)
-2 No answer/refused	-5 Missing; Not applicable for other reasons

SOCIETAL WELL-BEING

Q46. **Taking all things together, would you say you are** (*read out and code one answer*):
1 Very happy
2 Rather happy
3 Not very happy
4 Not at all happy

Q47. **All in all, how would you describe your state of health these days? Would you say it is…** (*read out*):
1 Very good
2 Good
3 Fair
4 Poor
5 Very poor

Q48. **Some people feel they have completely free choice and control over their lives, while other people feel that what they do has no real effect on what happens to them. Please use this scale where 1 means "no choice at all" and 10 means "a great deal of choice" to indicate how much freedom of choice and control you feel you have over the way your life turns out** (*code one number*):

No choice at all								A great deal of choice	
1	2	3	4	5	6	7	8	9	10

(*SHOW CARD 5*)
Q49. **All things considered, how satisfied are you with your life as a whole these days? Using this card on which 1 means you are "completely dissatisfied" and 10 means you are "completely satisfied" where would you put your satisfaction with your life as a whole?** (*Code one number*):

Completely dissatisfied								Completely satisfied	
1	2	3	4	5	6	7	8	9	10

(*SHOW CARD 6*)
Q50. **How satisfied are you with the financial situation of your household? Please use this card again to help with your answer** (*code one number*):

Completely dissatisfied								Completely satisfied	
1	2	3	4	5	6	7	8	9	10

In the last 12 months, how often have your or your family…?

		Often	Sometimes	Rarely	Never
Q51	Gone without enough food to eat	1	2	3	4
Q52	Felt unsafe from crime in your home	1	2	3	4
Q53	Gone without medicine or medical treatment that you needed	1	2	3	4
Q54	Gone without a cash income	1	2	3	4
Q55	Gone without a safe shelter over your head	1	2	3	4

Q56**. Comparing your standard of living with your parents' standard of living when they were about your age, would you say that you are better off, worse off or about the same?**
1. Better off,
2. Worse off,
3. Or about the same.

NG1. **What is your ethnic community, cultural group or tribe?** …………………………

		Very important	Quite important	Not so important	Not at all important
NG2.	**How important is your ethnic belonging to you?**	1	2	3	4

Coding for missing codes is as follows (do not read them and code only if the respondent mentions them him-/herself):
-1 Don't know -3 Not applicable (filter)
-2 No answer/refused -5 Missing; Not applicable for other reasons

SOCIAL CAPITAL, TRUST & ORGANIZATIONAL MEMBERSHIP

Q57. **Generally speaking, would you say that most people can be trusted or that you need to be very careful in dealing with people?** (*Code one answer*):

1 Most people can be trusted

2 Need to be very careful

I'd like to ask you how much you trust people from various groups. Could you tell me for each whether you trust people from this group completely, somewhat, not very much or not at all? (*Read out and code one answer for each*):

		Trust completely	Trust somewhat	Do not trust very much	Do not trust at all
Q58	Your family	1	2	3	4
Q59	Your neighborhood	1	2	3	4
Q60	People you know personally	1	2	3	4
Q61	People you meet for the first time	1	2	3	4
Q62	People of another religion	1	2	3	4
Q63	People of another nationality	1	2	3	4

I am going to name a number of organizations. For each one, could you tell me how much confidence you have in them: is it a great deal of confidence, quite a lot of confidence, not very much confidence or none at all? (*Read out and code one answer for each*):

		A great deal	Quite a lot	Not very much	None at all
Q64	The churches/mosques	1	2	3	4
Q65	The armed forces	1	2	3	4
Q66	The press	1	2	3	4
Q67	Television	1	2	3	4
Q68	Labor unions	1	2	3	4
Q69	The police	1	2	3	4
Q70	The courts	1	2	3	4
Q71	The government	1	2	3	4
Q72	Political parties	1	2	3	4
Q73	Parliament	1	2	3	4
Q74	The civil service	1	2	3	4
Q75	Universities	1	2	3	4
NG3	Hospitals	1	2	3	4
Q76	Elections	1	2	3	4
Q77	Major companies	1	2	3	4
Q78	Banks	1	2	3	4
Q79	Environmental organizations	1	2	3	4
Q80	Women's organizations	1	2	3	4
Q81	Charitable or humanitarian organizations	1	2	3	4
NG4	Community Leaders/Village elders	1	2	3	4
NG5	Religious leaders	1	2	3	4
NG6	Nearest health care facility	1	2	3	4
Q82	The African Union	1	2	3	4
Q83	The United Nations	1	2	3	4
Q84	The International Monetary Fund (IMF)	1	2	3	4
Q85	International Criminal Court (ICC)	1	2	3	4
Q86	The North Atlantic Treaty Organization (NATO)	1	2	3	4
Q87	The World Bank	1	2	3	4
Q88	The World Health Organization (WHO)	1	2	3	4
Q89	The World Trade Organization (WTO)	1	2	3	4

Coding for missing codes is as follows (do not read them and code only if the respondent mentions them him-/herself):

-1 Don't know -3 Not applicable (filter)

-2 No answer/refused -5 Missing; Not applicable for other reasons

(***SHOW CARD 7***)

Q90. Nowadays there's a lot of talk about international organizations. People sometimes say that international organizations should prioritize improving people's lives, even if this may imply that decisions are not made democratically. What do you think should international organizations prioritize, being effective or being democratic? If your views are somewhat mixed, choose the appropriate number in between.

Being effective								Being democratic	
1	2	3	4	5	6	7	8	9	10

(***SHOW CARD 8***)

Here are some questions about international organizations. Many people don't know the answers to these questions, but if you do please tell me.

		A	B	C
Q91	Five countries have permanent seats on the Security Council of the United Nations. Which ones of the following is not a member? *A) France, B) China, C) India*	1	2	3
Q92	Where are the headquarters of the International Monetary Fund (IMF) located? *A) Washington DC, B) London, C) Geneva*	1	2	3
Q93	Which of the following problems does the organization Amnesty International deal with? *A) Climate change, B) Human rights, C) Destruction of historic monuments*	1	2	3

Now I am going to read off a list of voluntary organizations. For each organization, could you tell me whether you are an active member, an inactive member or not a member of that type of organization? (*Read out and code one answer for each organization*):

		Active member	Inactive member	Don't belong
Q94	Church or religious organization	2	1	0
Q95	Sport or recreational organization, football/baseball/rugby team	2	1	0
Q96	Art, music or educational organization	2	1	0
Q97	Labor Union	2	1	0
Q98	Political party	2	1	0
Q99	Environmental organization	2	1	0
Q100	Professional association	2	1	0
Q101	Humanitarian or charitable organization	2	1	0
Q102	Consumer organization	2	1	0
Q103	Self-help group, mutual aid group	2	1	0
Q104	Women's group	2	1	0
Q105	Other organization	2	1	0

ECONOMIC VALUES

(***SHOW CARD 9***)

Now I'd like you to tell me your views on various issues. How would you place your views on this scale? 1 means you agree completely with the statement on the left; 10 means you agree completely with the statement on the right; and if your views fall somewhere in between, you can choose any number in between. (*Code one number for each issue*):

Q106 Incomes should be made more equal — There should be greater incentives for individual effort

1	2	3	4	5	6	7	8	9	10

Q107 Private ownership of business and industry should be increased — Government ownership of business and industry should be increased

1	2	3	4	5	6	7	8	9	10

Q108 Government should take more responsibility to ensure that everyone is provided for — People should take more responsibility to provide for themselves

1	2	3	4	5	6	7	8	9	10

Coding for missing codes is as follows (do not read them and code only if the respondent mentions them him-/herself):
-1 Don't know -3 Not applicable (filter)
-2 No answer/refused -5 Missing; Not applicable for other reasons

Q109 Competition is good — Competition is harmful

Competition is good									Competition is harmful
1	2	3	4	5	6	7	8	9	10

Q110 In the long run, hard work usually brings a better life — Hard work doesn't generally bring success—it's more a matter of luck and connections

In the long run, hard work usually brings a better life									Hard work doesn't generally bring success—it's more a matter of luck and connections
1	2	3	4	5	6	7	8	9	10

Q111. Here are two statements people sometimes make when discussing the environment and economic growth. Which of them comes closer to your own point of view? (*Read out and code one answer*):

1 Protecting the environment should be given priority, even if it causes slower economic growth and some loss of jobs.
2 Economic growth and creating jobs should be the top priority, even if the environment suffers to some extent.
3 Other answer (*code if volunteered only!*).

CORRUPTION

(***SHOW CARD 10***)

Q112. **Now I'd like you to tell me your views on corruption – when people pay a bribe, give a gift or do a favor to other people in order to get the things they need done or the services they need. How would you place your views on corruption in [your country] on a 10-point scale where "1" means "there is no corruption in [my country]" and "10" means "there is abundant corruption in [my country]". If your views are somewhat mixed, choose the appropriate number in between.**

There is no corruption in Nigeria									There is abundant corruption in Nigeria
1	2	3	4	5	6	7	8	9	10

Among the following groups of people, how many do you believe are involved in corruption? Tell me for each group if you believe it is none of them, few of them, most of them or all of them?

Interviewer: read out groups and code just one number for each of them!		None of them	Few of them	Most of them	All of them
Q113	State authorities	1	2	3	4
Q114	Business executives	1	2	3	4
Q115	Local authorities	1	2	3	4
Q116	Civil service providers (police, judiciary, civil servants, doctors, teachers)	1	2	3	4
Q117	Journalists and media	1	2	3	4

Q118. We want to know about your experience with local officials and service providers, like police officers, lawyers, doctors, teachers and civil servants in your community. How often do you think ordinary people like yourself or people from your neighbourhood have to pay a bribe, give a gift or do a favor to these people in order to get the services you need? Does it happen never, rarely, frequently or always? (*Interviewer: code just one number!*)

Never	Rarely	Frequently	Always
1	2	3	4

Q119. Can you tell me how strongly you agree or disagree with the following statement: "on the whole, women are less corrupt than men"?

1	2	3	4	0
Strongly agree	Agree	Disagree	Strongly disagree	Hard to say

(***SHOW CARD 11***)

Q120. **How high is the risk in this country being held accountable for giving or receiving a bribe, gift or favor in return for public service? To indicate your opinion, use a 10-point scale where "1" means "no risk at all" and "10" means "very high risk".** (*Interviewer: show card and code just one number!*)

No risk at all									Very high risk
1	2	3	4	5	6	7	8	9	10

Coding for missing codes is as follows (do not read them and code only if the respondent mentions them him-/herself):	
-1 Don't know	-3 Not applicable (filter)
-2 No answer/refused	-5 Missing; Not applicable for other reasons

MIGRATION

Q121. Now we would like to know your opinion about the people from other countries who come to live in Nigeria - the immigrants. How would you evaluate the impact of these people on the development of Nigeria?

Very good	Quite good	Neither good, nor bad	Quite bad	Rather bad
5	4	3	2	1

From your point of view, what have been the effects of immigration on the development of Nigeria? For each of the following statements about the effects of immigration, please, tell me whether you agree or disagree with it
(Interviewer, for every items code "agree" or "disagree". Don't read "hard to say", code only if mentioned by the respondent)

		Agree	Hard to say	Disagree
Q122	Fills important jobs vacancies	2	1	0
Q123	Strengthens cultural diversity	2	1	0
Q124	Increases the crime rate	2	1	0
Q125	Gives asylum to political refugees who are persecuted elsewhere	2	1	0
Q126	Increases the risks of terrorism	2	1	0
Q127	Offers people from poor countries a better living	2	1	0
Q128	Increases unemployment	2	1	0
Q129	Leads to social conflict	2	1	0

Q130. **How about people from other countries coming here to work. Which one of the following do you think the government should do?**

1. Let anyone come who wants to
2. Let people come as long as there are jobs available
3. Place strict limits on the number of foreigners who can come here
4. Prohibit people coming here from other countries

QNG7 **Many people also leave Nigeria for different reasons. How would you evaluate the impact of emigration <u>on the development of Nigeria?</u>**

Very good	Quite good	Neither good, nor bad	Quite bad	Rather bad
5	4	3	2	1

QNG8. **We would like to know why you think people leave Nigera for other countries. For each of the following statements about the effects of the emigration, please, tell me wheter you agree or disagree with it.**
(Interviewer, for every items code "agree" or "disagree". Don't read "hard to say", code only if mentioned by the respondent)

	Agree	Hard to say	Disagree
QNG8a. When young people leave it affects the future of Nigeria negatively	2	1	0
QNG8b. There will be more possibilities for the ones who stays	2	1	0
QNG8c. People leave because the elderly doesn't give the young ones any chances	2	1	0
QNG8d. People leave because they can earn more abroad	2	1	0
QNG8e. People leave because there's too much violence in the country	2	1	0
QNG8f. People leave because of environmental issues	2	1	0

SECURITY

Q131. Could you tell me how secure do you feel these days?

Very secure	1
Quite secure	2
Not very secure	3
Not at all secure	4

How frequently do the following things occur in your neighborhood?

	Very frequently	Quite frequently	Not frequently	Not at all frequently
Q132. Robberies	1	2	3	4
Q133. Alcohol consumption in the streets	1	2	3	4
Q134. Police or military interfere with people's private life	1	2	3	4
Q135. Racist behavior	1	2	3	4
Q136. Drug sale in streets	1	2	3	4
Q137. Street violence and fights	1	2	3	4
Q138. Sexual harassment	1	2	3	4

Which of the following things have you done for reasons of security? (MULTIPLE RESPONSE)

	Yes	No
Q139. Didn't carry much money	1	2
Q140. Preferred not to go out at night	1	2
Q141. Carried a knife, gun or other weapon	1	2

To what degree are you worried about the following situations?

	Very much	A good deal	Not much	Not at all
Q142. Losing my job or not finding a job	1	2	3	4
Q143. Not being able to give my children a good education	1	2	3	4

Have you been the victim of a crime during the past year?
And what about your immediate family – has someone in your family been the victim of a crime during the last year?

	Yes	No
Q144. Respondent	1	2
Q145. Family	1	2

To what degree are you worried about the following situations?

	Very much	A good deal	Not much	Not at all
Q146. A war involving my country	1	2	3	4
Q147. A terrorist attack	1	2	3	4
Q148. A civil war	1	2	3	4

Q149. **Most people consider both freedom and equality to be important, but if you had to choose between them, which one would you consider more important?**
1. Freedom
2. Equality

Q150. **Most people consider both freedom and security to be important, but if you had to choose between them, which one would you consider more important?**
1. Freedom
2. Security

Coding for missing codes is as follows (do not read them and code only if the respondent mentions them him-/herself):

-1 Don't know	-3 Not applicable (filter)
-2 No answer/refused	-5 Missing; Not applicable for other reasons

Q151. **Of course, we all hope that there will not be another war, but if it were to come to that, would you be willing to fight for your country?** (*Code one answer*):

1 Yes 2 No

POSTMATERIALIST INDEX

(*SHOW CARD 13*)

People sometimes talk about what the aims of this country should be for the next ten years. On this 'card are listed some of the goals which different people would give top priority.

Q152**. Would you please say which one of these you, yourself, consider the most important?** (*Code one answer only under "first choice"*)

Q153. **And which would be the next most important?** (*Code one answer only under "second choice"*)

	Q152 First choice	Q153 Second choice
1. A high level of economic growth	1	1
2. Making sure this country has strong defense forces	2	2
3. Seeing that people have more say about how things are done at their jobs and in their communities	3	3
4. Trying to make our cities and countryside (rural areas) more beautiful	4	4

(*SHOW CARD 14*)

Q154. **If you had to choose, which one of the things on this card would you say is most important?** (*Code one answer only under "first choice"*):

Q155. **And which would be the next most important?** (*Code one answer only under "second choice"*):

	Q154 First choice	Q155 Second choice
1. Maintaining order in the nation	1	1
2. Giving people more say in important government decisions	2	2
3. Fighting rising prices	3	3
4. Protecting freedom of speech	4	4

(*SHOW CARD 15*)

Q156. **Here is another list. In your opinion, which one of these is most important?** (*Code one answer only under "first choice"*):

Q157. **And what would be the next most important?** (*Code one answer only under "second choice"*):

	Q156 First choice	Q157 Second choice
1. A stable economy	1	1
2. Progress toward a less impersonal and more humane society	2	2
3. Progress toward a society in which ideas count more than money	3	3
4. The fight against crime	4	4

Coding for missing codes is as follows (do not read them and code only if the respondent mentions them him-/herself):

-1 Don't know	-3 Not applicable (filter)
-2 No answer/refused	-5 Missing; Not applicable for other reasons

SCIENCE & TECHNOLOGY

(*SHOW CARD 16*)
Now, I would like to read some statements and ask how much you agree or disagree with each of these statements. For these questions, a 1 means that you "completely disagree" and a 10 means that you "completely agree." (*Code one number for each statement*):

Q158. **Science and technology are making our lives healthier, easier, and more comfortable.**

Completely disagree									Completely agree
1	2	3	4	5	6	7	8	9	10

Q159. **Because of science and technology, there will be more opportunities for the next generation.**

Completely disagree									Completely agree
1	2	3	4	5	6	7	8	9	10

Q160. **We depend too much on science and not enough on faith.**

Completely disagree									Completely agree
1	2	3	4	5	6	7	8	9	10

Q161. **One of the bad effects of science is that it breaks down people's ideas of right and wrong.**

Completely disagree									Completely agree
1	2	3	4	5	6	7	8	9	10

Q162. **It is not important for me to know about science in my daily life.**

Completely disagree									Completely agree
1	2	3	4	5	6	7	8	9	10

Q163. **All things considered, would you say that the world is better off, or worse off, because of science and technology? Please tell me which comes closest to your view on this scale: 1 means that "the world is a lot worse off," and 10 means that "the world is a lot better off."** (*Code one number*):

A lot worse off									A lot better off
1	2	3	4	5	6	7	8	9	10

Coding for missing codes is as follows (do not read them and code only if the respondent mentions them him-/herself):
-1 Don't know -3 Not applicable (filter)
-2 No answer/refused -5 Missing; Not applicable for other reasons

RELIGIOUS VALUES

(*SHOW CARD 17*)

Q164. **How important is God in your life? Please use this scale to indicate. 10 means "very important" and 1 means "not at all important."** (*Code one number*):

Not at all important									Very important
1	2	3	4	5	6	7	8	9	10

Which, if any, of the following do you believe in?

		Yes	No
Q165	God	1	2
Q166	Life after death	1	2
Q167	Hell	1	2
Q168	Heaven	1	2

Please tell us if you strongly agree, agree, disagree, or strongly disagree with the following statements:

		Strongly agree	Agree	Disagree	Strongly disagree
Q169	Whenever science and religion conflict, religion is always right	1	2	3	4
Q170	The only acceptable religion is my religion.	1	2	3	4

(*SHOW CARD 18*)

Q171. **Apart from weddings and funerals, about how often do you attend religious services these days?** (*Code one answer*):

1 More than once a week
2 Once a week
3 Once a month
4 Only on special holy days
5 Once a year
6 Less often
7 Never, practically never

(*SHOW CARD 19)*

Q172. **Apart from weddings and funerals, about how often do you pray?** (*Code one answer*):

1 Several times a day
2 Once a day
3 Several times each week
4 Only when attending religious services
5 Only on special holy days
6 Once a year
7 Less often
8 Never, practically never

Q173. **Independently of whether you attend religious services or not, would you say you are…?** (*read out and code one answer*):

1 A religious person
2 Not a religious person
3 An atheist

Q174. **With which one of the following statements do you agree most? The basic meaning of religion is:**

1. To follow religious norms and ceremonies 2. To do good to other people

Q175. **And with which of the following statements do you agree most? The basic meaning of religion is:**

1. To make sense of life after death 2. To make sense of life in this world

Coding for missing codes is as follows (do not read them and code only if the respondent mentions them him-/herself):
-1 Don't know -3 Not applicable (filter)
-2 No answer/refused -5 Missing; Not applicable for other reasons

ETHICAL VALUES AND NORMS

Q176. **How much do you agree or disagree with the statement that nowadays one often has trouble deciding which moral rules are the right ones to follow?**

Completely agree									Completely disagree
1	2	3	4	5	6	7	8	9	10

(*SHOW CARD 20*)
Please tell me for each of the following actions whether you think it can always be justified, never be justified, or something in between, using this card. (*Read out and code one answer for each statement*):

		Never justifiable									Always justifiable
Q177	Claiming government benefits to which you are not entitled	1	2	3	4	5	6	7	8	9	10
Q178	Avoiding a fare on public transport	1	2	3	4	5	6	7	8	9	10
Q179	Stealing property	1	2	3	4	5	6	7	8	9	10
Q180	Cheating on taxes if you have a chance	1	2	3	4	5	6	7	8	9	10
Q181	Someone accepting a bribe in the course of their duties	1	2	3	4	5	6	7	8	9	10
Q182	Homosexuality	1	2	3	4	5	6	7	8	9	10
Q183	Prostitution	1	2	3	4	5	6	7	8	9	10
Q184	Abortion	1	2	3	4	5	6	7	8	9	10
Q185	Divorce	1	2	3	4	5	6	7	8	9	10
Q186	Sex before marriage	1	2	3	4	5	6	7	8	9	10
NG9	To use contraceptives	1	2	3	4	5	6	7	8	9	10
NG10	To have more than one wife at the same time	1	2	3	4	5	6	7	8	9	10
Q187	Suicide	1	2	3	4	5	6	7	8	9	10
Q188	Euthanasia (assisted dying)	1	2	3	4	5	6	7	8	9	10
Q189	For a man to beat his wife	1	2	3	4	5	6	7	8	9	10
Q190	Parents beating children	1	2	3	4	5	6	7	8	9	10
Q191	Violence against other people	1	2	3	4	5	6	7	8	9	10
Q192	Terrorism as a political, ideological or religious mean	1	2	3	4	5	6	7	8	9	10
Q193	Having casual sex	1	2	3	4	5	6	7	8	9	10
Q194	Political violence	1	2	3	4	5	6	7	8	9	10
NG11	Any form of violence	1	2	3	4	5	6	7	8	9	10
Q195	Death Penalty	1	2	3	4	5	6	7	8	9	10

Do you think that the Nigeria government should or should not have the right to do the following:

		Definitely should have the right	Probably should have the right	Probably should not have the right	Definitely should not have the right
Q196	Keep people under video surveillance in public areas	1	2	3	4
Q197	Monitor all e-mails and any other information exchanged on the Internet	1	2	3	4
Q198	Collect information about anyone living in Nigeria without their knowledge	1	2	3	4

Coding for missing codes is as follows (do not read them and code only if the respondent mentions them him-/herself):
-1 Don't know -3 Not applicable (filter)
-2 No answer/refused -5 Missing; Not applicable for other reasons

POLITICAL INTEREST & POLITICAL PARTICIPATION

Q199. **How interested would you say you are in politics? Are you** (*read out and code one answer*):
1 Very interested
2 Somewhat interested
3 Not very interested
4 Not at all interested

Q200. **When you get together with your friends, would you say you discuss political matters frequently, occasionally or never?**
1 Frequently
2 Occasionally
3 Never

People learn what is going on in this country and the world from various sources. For each of the following sources, please indicate whether you use it to obtain information daily, weekly, monthly, less than monthly or never (*read out and code one answer for each*):

	Daily	Weekly	Monthly	Less than monthly	Never
Q201. Daily newspaper	1	2	3	4	5
Q202. TV news	1	2	3	4	5
Q203. Radio news	1	2	3	4	5
Q204. Mobile phone	1	2	3	4	5
Q205. Email	1	2	3	4	5
Q206. Internet	1	2	3	4	5
Q207. Social media (Facebook, Twitter, etc.)	1	2	3	4	5
Q208. Talk with friends or colleagues	1	2	3	4	5

(*SHOW CARD 21*)
Now I'd like you to look at this card. I'm going to read out some forms of political action that people can take, and I'd like you to tell me, for each one, whether you have done any of these things, whether you might do it or would never under any circumstances do it (*read out and code one answer for each action*):

	Have done	Might do	Would never do
Q209. Signing a petition	1	2	3
Q210. Joining in boycotts	1	2	3
Q211. Attending peaceful demonstrations	1	2	3
Q212. Joining strikes	1	2	3

(*SHOW CARD 22*)
What about these forms of political action and social activism that people can take? Please, tell me for each of them if you have done any of these things, whether you might do it or would never under any circumstances do it (*read out and code one answer for each action*):

	Have done	Might do	Would never do	Not applicable
Q213. Donating to a group or campaign	1	2	3	-3
Q214. Contacting a government official	1	2	3	-3
Q215. Encouraging others to take action about political issues	1	2	3	-3
Q216. Encouraging others to vote	1	2	3	-3

Coding for missing codes is as follows (do not read them and code only if the respondent mentions them him-/herself):
-1 Don't know　　-3 Not applicable (filter)
-2 No answer/refused　　-5 Missing; Not applicable for other reasons

(*SHOW CARD 23*)
Now I'd like you to look at this card. I'm going to read out some other forms of political action that people can take <u>using Internet and social media tools like Facebook, Twitter etc.</u>, and I'd like you to tell me, for each one, whether you have done any of these things, whether you might do it or would never under any circumstances do it (*read out and code one answer for each action; if the respondent does not use Internet and social media, please, code "-3"=not applicable*):

		Have done	Might do	Would never do	Not applicable
Q217	Searching information about politics and political events	1	2	3	-3
Q218	Signing an electronic petition	1	2	3	-3
Q219	Encouraging other people to take any form of political action	1	2	3	-3
Q220	Organizing political activities, events, protests	1	2	3	-3

When elections take place, do you vote always, usually or never? Please tell me separately for each of the following levels (*Read out and code one answer for each item*):

	Always	Usually	Never	Not allowed to vote
Q221. Local level	1	2	3	4
Q222. National level	1	2	3	4

(SHOW CARD 24)
Q223. If there were a national election tomorrow, for which party on this list would you vote? Just call out the number on this card. ***If DON'T KNOW: Which party appeals to you most?***

People Democratic Party (PDP)	1
All Progressives Congress (APC)	2
All Progressive Grand Alliance (APGA)	3
Justice Party (JP)	4
Peoples Mandate Party (PMP)	5
Green Party of Nigeria (GPN)	6
National Democratic Party (NDP)	7
United Nigeria Peoples Party (UNPP)	8
National Action Council (NAC)	9
African Renaissance Party (ARP)	10
Democratic Alternative (DA)	11
Peoples Redemption Party (PRP)	12
Nigeria Advance Party (NAP)	13
Liberial Democratic Party of Nigeria (LDPN)	14
Progressive Action Congress (PAC)	15
Movement for Democracy and Justice (MDJ)	16
National Conscience Party (NCP)	17
Alliance for Democracy (AD)	18
People Progressive Alliance (PPA)	20
Accord Party (AP)	21
Fresh Party (FP)	22
Africa Democratic Coalition (ADC)	23
Other (specify)	97
Don't know	-1
Refused	-2

Coding for missing codes is as follows (do not read them and code only if the respondent mentions them him-/herself):
-1 Don't know -3 Not applicable (filter)
-2 No answer/refused -5 Missing; Not applicable for other reasons

In your view, how often do the following things occur in this country's elections?

	Very often	Fairly often	Not often	Not at all often
Q224. Votes are counted fairly	1	2	3	4
Q225. Opposition candidates are prevented from running	1	2	3	4
Q226. TV news favors the governing party	1	2	3	4
Q227. Voters are bribed	1	2	3	4
Q228. Journalists provide fair coverage of elections	1	2	3	4
Q229. Election officials are fair	1	2	3	4
Q230. Rich people buy elections	1	2	3	4
Q231. Voters are threatened with violence at the polls	1	2	3	4
Q232. Voters are offered a genuine choice in the elections	1	2	3	4
Q233. Women have equal opportunities to run for office	1	2	3	4

Q234. **Some people think that having honest elections makes a lot of difference in their lives; other people think that it doesn't matter much. How important would you say is having honest elections for you—very important, rather important, not very important or not at all important?**

1. Very important
2. Rather important
3. Not very important
4. Not at all important

POLITICAL CULTURE & POLITICAL REGIMES

I'm going to describe various types of political systems and ask what you think about each as a way of governing this country. For each one, would you say it is a very good, fairly good, fairly bad or very bad way of governing this country? (*Read out and code one answer for each*):

		Very good	Fairly good	Fairly bad	Very bad
Q235	Having a strong leader who does not have to bother with parliament (the legislature, federal executive council) and elections	1	2	3	4
Q236	Having experts, not government, make decisions according to what they think is best for the country	1	2	3	4
Q237	Having the army rule	1	2	3	4
Q238	Having a democratic political system	1	2	3	4
Q239	Having a system governed by religious law in which there are no political parties or elections	1	2	3	4

(*SHOW CARD 25*)

Q240. **In political matters, people talk of "the left" and "the right." How would you place your views on this scale, generally speaking?** (*Code one number*):

Left									Right
1	2	3	4	5	6	7	8	9	10

Coding for missing codes is as follows (do not read them and code only if the respondent mentions them him-/herself):
-1 Don't know -3 Not applicable (filter)
-2 No answer/refused -5 Missing; Not applicable for other reasons

(*SHOW CARD 26*)

Many things are desirable, but not all of them are essential characteristics of democracy. Please tell me for each of the following things how essential you think it is as a characteristic of democracy. Use this scale where 1 means "not at all an essential characteristic of democracy" and 10 means it definitely is "an essential characteristic of democracy" (*read out and code one answer for each*):

	['It is against democracy' - Interviewer: Do not read this and code only if mentioned by the respondent him-/herself]	*It is against demo-cracy*	Not an essential characteristic of democracy								An essential characteristic of democracy
Q241	Governments tax the rich and subsidize the poor.	*0*	1	2	3	4	5	6	7	8	9	10
Q242	Religious authorities ultimately interpret the laws.	*0*	1	2	3	4	5	6	7	8	9	10
Q243	People choose their leaders in free elections.	*0*	1	2	3	4	5	6	7	8	9	10
Q244	People receive state aid for unemployment.	*0*	1	2	3	4	5	6	7	8	9	10
Q245	The army takes over when government is incompetent.	*0*	1	2	3	4	5	6	7	8	9	10
Q246	Civil rights protect people from state oppression.	*0*	1	2	3	4	5	6	7	8	9	10
Q247	The state makes people's incomes equal.	*0*	1	2	3	4	5	6	7	8	9	10
Q248	People obey their rulers.	*0*	1	2	3	4	5	6	7	8	9	10
Q249	Women have the same rights as men.	*0*	1	2	3	4	5	6	7	8	9	10

(*SHOW CARD 27*)

Q250. **How important is it for you to live in a country that is governed democratically? On this scale where 1 means it is "not at all important" and 10 means "absolutely important" what position would you choose?** (*Code one number*):

Not at all important									Absolutely important
1	2	3	4	5	6	7	8	9	10

(*SHOW CARD 28*)

Q251. **And how democratically is this country being governed today? Again using a scale from 1 to 10, where 1 means that it is "not at all democratic" and 10 means that it is "completely democratic," what position would you choose?** (*Code one number*):

Not at all democratic									Completely democratic
1	2	3	4	5	6	7	8	9	10

Q252. **On a scale from 1 to 10 where "1" is "not satisfied at all" and "10" is "completely satisfied", how satisfied are you with how the political system is functioning in your country these days?**

Not satisfied at all									Completely satisfied
1	2	3	4	5	6	7	8	9	10

Q253. **How much respect is there for individual human rights nowadays in Nigeria? Do you feel there is** (*read out and code one answer*):

1 A great deal of respect for individual human rights
2 Fairly much respect
3 Not much respect
4 No respect at all

Coding for missing codes is as follows (do not read them and code only if the respondent mentions them him-/herself):
-1 Don't know -3 Not applicable (filter)
-2 No answer/refused -5 Missing; Not applicable for other reasons

Q254. **How proud are you to be Nigerian?** (*Read out and code one answer*):

1 Very proud
2 Quite proud
3 Not very proud
4 Not at all proud
5 I am not [country's nationality] (*do not read out! Code only if volunteered!*)

(***SHOW CARD 29***)

People have different views about themselves and how they relate to the world. Using this card, would you tell me how close do you feel to…? (*Read out and code one answer for each statement*):

	Very close	Close	Not very close	Not close at all
Q255. Village, town or city	1	2	3	4
Q256. Your region or State [county, region, district]	1	2	3	4
Q257. Nigeria [Country]	1	2	3	4
Q258. Africa [Continent; e.g. Europe, Asia etc.]	1	2	3	4
Q259. World	1	2	3	4

Coding for missing codes is as follows (do not read them and code only if the respondent mentions them him-/herself):

-1 Don't know	-3 Not applicable (filter)
-2 No answer/refused	-5 Missing; Not applicable for other reasons

DEMOGRAPHICS

Q260. **Respondent's sex** (***Code respondent's sex by observation, don't ask about it!***):

1 Male
2 Female

Q261. **Can you tell me your year of birth, please?** [ ][ ][ ][ ]

Q262. **This means you are _______ years old** (*write in age in two digits*).
Q263. **Were you born in this country or are you an immigrant to this country?** *(Code one answer)*

Q264-265. **Are your mother and father immigrants to this country or not? Please, indicate separately for each of them** (*read out and code one answer for each*):

Q266-268. **In which country were you, your father and your mother (or those who raised you) born?** *(Interviewer, write the country name as stated by the respondent and use the list of codes to code the country later, after the interview is completed)*

	Q263. Respondent	Q264. Mother of the respondent	Q265. Father of the respondent
1. Born in Nigeria	1	1	1
2. Born outside Nigeria	2	2	2

	Q266. Respondent	Q267. Mother of the respondent	Q268. Father of the respondent
Country of birth: write in the name of country	__________	__________	__________
CODE the country of birth	[ ][ ][ ][ ]	[ ][ ][ ][ ]	[ ][ ][ ][ ]

Q269. **Are you a citizen of this country?**

1 Yes, I am a citizen of this country.
2 Not, I am not a citizen of this country.

Q270. **Including yourself, how many people – including children – live here regularly as members of this household?**

____

NG10. **Do you live in the same area where you were born?**

1 Yes 2 No

Q271**. Do you live with your parents or your parents in law? (*Code one answer*):**

1. No
2. Yes, own parent(s)
3. Yes, parent(s) in law
4. Yes, both own parent(s) and parent(s) in law

Q272. **What language do you normally speak at home?** (*Code one answer!*)

English	1	Tiv	6
Pidgin English	2	Urhobo	7
Yoruba	3	Isoko	8
Igbo	4	Itsekiri	9
Hausa	5	Others [Specify]	10
Tiv	6	__________________	

Coding for missing codes is as follows (do not read them and code only if the respondent mentions them him-/herself):
-1 Don't know -3 Not applicable (filter)
-2 No answer/refused -5 Missing; Not applicable for other reasons

Q273. **Are you currently** (*read out and code one answer only*):

1 Married
2 Living together as married
3 Divorced
4 Separated
5 Widowed
6 Single

Q274. **Do you have any children?** (*Code 0 if no, and respective number if yes*): _____________

If you are a mother or to your knowledge a father of at least one child please anser the following question, if not go on to question 275.

If you are a man please answer this question:
NG11. What was the age of your first child's mother when she gave birth for the very first time ever? …………………..

If you are a woman, please anser this question:
NG12. What was your own age when you gave birth to your first child ………………………

Q275-278. **What is the highest educational level that you, your spouse, your mother and your father have attained?**[1]
[Interviewer: code for each person separately. The table below uses codes ISCED-2011 – International Standard Classification for Education used by the UN and UNESCO. Your supervisor will provide you with a national-adapted list of codes. If the respondent has no spouse, no father or no mother, code "-3"=not applicable
Note, 'completed' = diploma or certificate

		Q275. Respondent	Q276. Spouse	Q277. Mother	Q278. Father
0	Early childhood education (ISCED 0) / no education	0	0	0	0
1	Primary education (ISCED 1)	1	1	1	1
2	Lower secondary education (ISCED 2)/Junior secondary school	2	2	2	2
3	Upper secondary education (ISCED 3)/ senior secondary school	3	3	3	3
4	Post-secondary non-tertiary education (ISCED 4)/Technical school	4	4	4	4
5	Short-cycle tertiary education (ISCED 5)/OND/NCE/Nursing	5	5	5	5
6	Bachelor or equivalent (ISCED 6)/HND	6	6	6	6
7	Master or equivalent (ISCED 7)	7	7	7	7
8	Doctoral or equivalent (ISCED 8)	8	8	8	8
	For DK/ NA & other codes	___________	________	_________	_______

[1] Dear national team leader, when localizing the questionnaire, conducting the survey and doing data entry, please, introduce 2 variables per each person mentioned in Q274-Q277 as following: Q274-Education of the respondent according to ISCED and Q274A-Education of the respondent according to the classification of your national system of education and so on for the spouse, mother and father of the respondent. Please, make sure you have provided then the list of national education codes to the WVSA Secretariat and Data archive.

Coding for missing codes is as follows (do not read them and code only if the respondent mentions them him-/herself):
-1 Don't know -3 Not applicable (filter)
-2 No answer/refused -5 Missing; Not applicable for other reasons

Please, code the levels of education according to the existing in Nigeria classification of education levels:

	Q275A Respondent	Q276A Spouse	Q277A Mother	Q278A Father
No formal education	0	0	0	0
Incomplete primary school	1	1	1	1
Complete primary school	2	2	2	2
Incomplete secondary school: technical/vocational type	3	3	3	3
Complete secondary school: technical/vocational type	4	4	4	4
Incomplete secondary: university-preparatory type	5	5	5	5
Complete secondary: university-preparatory type	6	6	6	6
Some university-level education, without degree	7	7	7	7
University-level education, with degree	8	8	8	8
Kuranic school	9	9	9	9
Others **[Specify]**	10	10	10	10
Don't know	-1	-1	-1	-1
No answer/refused	-2	-2	-2	-2

Q279. **Are you employed now or not? If yes, about how many hours a week? If more than one job: only for the main job** (*code one answer*):

Q280. **Is your spouse employed? In his/her LAST job was he/she employed (either full time or part time) or was he/she self-employed?**

	Q279. Respondent	Q280. Spouse
Yes, has paid employment:		
Full time employee (30 hours a week or more)	1	1
Part time employee (less than 30 hours a week)	2	2
Self employed	3	3
No, no paid employment:		
Retired/pensioned	4	4
Housewife not otherwise employed	5	5
Student	6	6
Unemployed	7	7
Other (*write in*):______________________	8	8

Q281. **To which of the following occupational groups do you belong?** *Interviewer: ask about respondent's last job if he/she does not have a job now*

Q282**. To which of the following occupational groups does your spouse belong?** *Interviewer: ask about the last job if he/she does not have a job now*

Q283. **When you were 14, to which of the following occupational groups did your father belong?** *Interviewer: ask about father's last job if he did not have a job or had already died when the respondent was 14 years' old*

		Q281. Respondent	Q282. Spouse	Q283. Father
1	Professional and technical (for example: doctor, teacher, engineer, artist, accountant, nurse)	1	1	1
2	Higher administrative (for example: banker, executive in big business, high government official, union official)	2	2	2
3	Clerical (for example: secretary, clerk, office manager, civil servant, bookkeeper	3	3	3
4	Sales (for example: sales manager, shop owner, shop assistant, insurance	4	4	4

Coding for missing codes is as follows (do not read them and code only if the respondent mentions them him-/herself):
-1 Don't know -3 Not applicable (filter)
-2 No answer/refused -5 Missing; Not applicable for other reasons

	agent, buyer)			
5	Service (for example: restaurant owner, police officer, waitress, barber, caretaker)	5	5	5
6	Skilled worker (for example: foreman, motor mechanic, printer, seamstress, tool and die maker, electrician)	6	6	6
7	Semi-skilled worker (for example: bricklayer, bus driver, cannery worker, carpenter, sheet metal worker, baker)	7	7	7
8	Unskilled worker (for example: laborer, porter, unskilled factory worker, cleaner)	8	8	8
9	Farm worker (for example: farm laborer, tractor driver)	9	9	9
10	Farm proprietor, farm manager	10	10	10
0	Never had a job	0	0	0

Q284. **Are you working for the government or public institution, for private business or industry, or for a private non-profit organization? If you do not work currently, characterize your major work in the past! Do you or did you work for** (*read out and code one answer*):

1 Government or public institution
2 Private business or industry
3 Private non-profit organization

Q285**. Are you the chief wage earner in your household?** (*Code one answer*):

1 Yes
2 No

Q286. **During the past year, did your family** (*read out and code one answer*):

1 Save money
2 Just get by
3 Spent some savings
4 Spent savings and borrowed money

Q287**. People sometimes describe themselves as belonging to the working class, the middle class, or the upper or lower class. Would you describe yourself as belonging to the** (*read out and code one answer*):

1 Upper class
2 Upper middle class
3 Lower middle class
4 Working class
5 Lower class

(***SHOW CARD 30***)

Q288. **On this card is an income scale on which 1 indicates the lowest income group and 10 the highest income group in your country. We would like to know in what group your household is. Please, specify the appropriate number, counting all wages, salaries, pensions and other incomes that come in.** (*Code one number*):

Lowest group									Highest group
1	2	3	4	5	6	7	8	9	10

Coding for missing codes is as follows (do not read them and code only if the respondent mentions them him-/herself):

-1 Don't know	-3 Not applicable (filter)
-2 No answer/refused	-5 Missing; Not applicable for other reasons

Q289. **Do you belong to a religion or religious denomination? If yes, which one?** (Code answer due to list below. Code 0, if respondent answers to have no denomination!)

No:	do not belong to a denomination	0
Yes:	Roman Catholic	1
	Protestant	2
	Orthodox (Russian/Greek/etc.)	3
	Jew	4
	Muslim	5
	Hindu	6
	Buddhist	7
	Other (*write in*):_____________	8

[NOTE: If your own society does not fit into this coding system, please devise an alternative, following this as closely as possible; for example, in Islamic countries, ask about Sunni, Shia, etc. Send a list of the categories used here along with your data].

Q290. Ethnic group of the respondent:

Yoruba	1
Hausa	2
Igbo	3
Fulani	4
Tiv	5
Ibibio	6
Other Africans	7
Asians	8
European	9
Others **[Write in]**____________________	10
**WRITE IN COUNTRY OF ORIGIN**________________________	11

Coding for missing codes is as follows (do not read them and code only if the respondent mentions them him-/herself):
-1 Don't know -3 Not applicable (filter)
-2 No answer/refused -5 Missing; Not applicable for other reasons

GENDER NORMS AND FAMILY PLANNING

(***SHOW CARD 31***)

I am now going to read out a few statements, and I would like you to think about the role of men and women in the community where you live. In general, who usually decides about the following things. 1 means that men usually decide and 9 means women that women usually decide – 5 means that men and women decide equally. (*Code one number for each statement*): (Instruction to interviewer).

Q291. **How earnings will be used**

Men				Both Equally				Women
1	2	3	4	5	6	7	8	9

Q292. **Major household purchases**

Men				Both Equally				Women
1	2	3	4	5	6	7	8	9

Q293. **Health care visits and spending**

Men				Both Equally				Women
1	2	3	4	5	6	7	8	9

Q294. **Whether a woman should give birth at a clinic**

Men				Both Equally				Women
1	2	3	4	5	6	7	8	9

Q295. **Care for children's health**

Men				Both Equally				Women
1	2	3	4	5	6	7	8	9

Q296. **Visit to family or relatives**

Men				Both Equally				Women
1	2	3	4	5	6	7	8	9

Q297. **Whether girls should go to school**

Men				Both Equally				Women
1	2	3	4	5	6	7	8	9

Q298. **When girls should marry**

Men				Both Equally				Women
1	2	3	4	5	6	7	8	9

Q299. **With whom girls should marry**

Men				Both Equally				Women
1	2	3	4	5	6	7	8	9

Q300. **If, and when to have children**

Men				Both Equally				Women
1	2	3	4	5	6	7	8	9

Q301**. Number of children**

Men				Both Equally				Women
1	2	3	4	5	6	7	8	9

Q302. **If, and when to have sex**

Men				Both Equally				Women
1	2	3	4	5	6	7	8	9

Q303. **Whether to use condoms**

Men				Both Equally				Women
1	2	3	4	5	6	7	8	9

Q304. **Whether to use other modern contraceptives**

Men				Both Equally				Women
1	2	3	4	5	6	7	8	9

Q305. **If girls should be circumcised**

Men				Both Equally				Women
1	2	3	4	5	6	7	8	9

Q306. **If boys should be circumcised**

Men				Both Equally				Women
1	2	3	4	5	6	7	8	9

Coding for missing codes is as follows (do not read them and code only if the respondent mentions them him-/herself):

-1 Don't know	-3 Not applicable (filter)
-2 No answer/refused	-5 Missing; Not applicable for other reasons

Please tell us if you strongly agree, agree, disagree, or strongly disagree with the following statements:

		Strongly agree	Agree	Disagree	Strongly disagree
Q307	Girls and women should themselves decide when, if and with whom they should marry	1	2	3	4
Q308	A girl should wait to marry until she has completed secondary school.	1	2	3	4
Q309	A boy should wait to marry until he has completed secondary school.	1	2	3	4
Q310	It is important for girls to continue their schooling even if they become pregnant and have children	1	2	3	4
Q311	A girl is ready for marriage once she starts menstruating.	1	2	3	4
Q312	Marrying girls young can help provide them security.	1	2	3	4
Q313	Even if a girl does not want to be married, she should honour the decisions/ wishes of her family.	1	2	3	4
Q314	Even if a boy does not want to be married, he should honour the decisions/ wishes of his family.	1	2	3	4
Q315	A girl should wait to have children until she is at least 18 years old.	1	2	3	4
Q316	A boy should wait to have children until he is at least 18 years old.	1	2	3	4
Q317	It is important for a woman to have children as soon as possible after she has married.	1	2	3	4
Q318	It is important for a man to have children as soon as possible after he has married.	1	2	3	4
Q319	It is safer for a woman to give birth at a clinic than at home.	1	2	3	4
Q320	Women should have access to safe abortion services.	1	2	3	4
Q321	A woman should be in love with someone before having sex with that person.	1	2	3	4
Q322	A man should be in love with someone before having sex with that person.	1	2	3	4
Q323	Women who carry condoms on them are easy.	1	2	3	4
Q324	Men should be outraged if their wife/partner asks them to use a condom.	1	2	3	4
Q325	It is a woman's responsibility to avoid getting pregnant.	1	2	3	4
Q326	Only when a woman has a child is she a real woman.	1	2	3	4
Q327	A real man produces a male child.	1	2	3	4
Q328	Having a son is always better than having a daughter.	1	2	3	4
Q329	A couple should decide together if they want to have children.	1	2	3	4
Q330	A man and a woman should decide together whether to use contraceptives.	1	2	3	4
Q331	Contraceptives should be available for everyone, whether or not one is married.	1	2	3	4
Q332	Sexuality education promotes sexual activity among young people.	1	2	3	4

Coding for missing codes is as follows (do not read them and code only if the respondent mentions them him-/herself):
-1 Don't know -3 Not applicable (filter)
-2 No answer/refused -5 Missing; Not applicable for other reasons

Q333a. *For women*

Some women feel they have completely free choice and control over family planning (if, when and how many children to have), while others don't. Please use this scale where 1 means "no choice at all" and 10 means "a great deal of choice" to indicate how much freedom of choice and control you feel that you have over your family planning. (*code one number*):

No choice at all									A great deal of choice
1	2	3	4	5	6	7	8	9	10

Q333b. *For men*

Some men feel they have completely free choice and control over family planning (if, when and how many children to have), while others don't. Please use this scale where 1 means "no choice at all" and 10 means "a great deal of choice" to indicate how much freedom of choice and control you feel that you have over your family planning. (*code one number*):

No choice at all									A great deal of choice
1	2	3	4	5	6	7	8	9	10

Now, we would like to ask you a bit more about contraception and family planning

Q334a. Have you ever heard of contraceptive methods?

A great deal	Quite a lot	Not very much	Not at all
1	2	3	4

Q334b. Which of the following contraceptive methods have you head about (TICK ALL THAT APPLY)

Method of contraceptive	Heard about
1. Injectable (Depo, Norigynon etc)	☐
2. Pill	☐
3. Emergency contraception	☐
4. Implant i.e. Jadelle, Implanon	☐
5. Bilateral Tubal ligation	☐
6. Lactation Amenorrhoea (breastfeeding)	☐
7. Male condom alone	☐
8. Female condom alone	☐
9. My spouse/partner did vasectomy	☐
10. IUD	☐
11. Diaphragm	☐
12. Foam/Jelly	☐
13. Withdrawal	☐
14. Safe periods	☐
15. Others (Specify)_______________	☐
16. Don't want to answer/Don't know	☐

Q 337. **In general, how motivated do you think women in your community are to use modern contraceptives?**

A great deal	Quite a lot	Not very much	Not at all
1	2	3	4

Q 337. **In general, how motivated do you think women in your community are to use modern contraceptives? (including supporting their female partners to do so)?**

A great deal	Quite a lot	Not very much	Not at all
1	2	3	4

Coding for missing codes is as follows (do not read them and code only if the respondent mentions them him-/herself):

-1 Don't know	-3 Not applicable (filter)
-2 No answer/refused	-5 Missing; Not applicable for other reasons

Q338a. **How easy is modern contraceptives to access for women in your community?**

Very easy	Quite easy	Not very easy	Not at all easy
1	2	3	4

Q338b. **Is it acceptable for women to use contraceptives in your community and neigbourhood?**

Totally acceptable	Quite acceptable	Not very acceptable	Not at all acceptable
1	2	3	4

Q339. **I would like to ask a question about your last birth (or current) pregnancy**.
At the time you became pregnant, did you want to become pregnant then, or did you want to wait until later, or did you not want to have any (more) children at all?

1. I wanted to become pregnant then	☐
2. I wanted to become pregnant later	☐
3. I did not want to become pregnant at all	☐
4. Don't know/don't want to answer	☐

I am now going to name a number of different health care providers. For each provider, please tell me how much confidence you have in them when it comes to family planning and child birth: is it a great deal of confidence, quite a lot of confidence, not very much confidence or none at all? (*Read out and code one answer for each*):

		A great deal	Quite a lot	Not very much	None at all
Q340	Doctor	1	2	3	4
Q341	Nurse	1	2	3	4
Q342	Auxiliary Nurse	1	2	3	4
Q343	Midwife (at the clinic)	1	2	3	4
Q344	Family planning counselor	1	2	3	4
Q345	Community health worker	1	2	3	4
Q346	Traditional birth attendant	1	2	3	4
Q347	Traditional healer	1	2	3	4
Q348	Religious leader (imam, church, priest, faith healers etc)	1	2	3	4
Q349	Youth clinic or center	1	2	3	4
Q350	Family member	1	2	3	4
Q351	Other ……..	1	2	3	4

How much confidence do you have in the health services/clinic/nearest hospital to deliver the following: is it a great deal of confidence, quite a lot of confidence, not very much confidence or none at all? (*Read out and code one answer for each*):

		A great deal	Quite a lot	Not very much	None at all
Q352	Safe contraceptives	1	2	3	4
Q353	Family planning counseling	1	2	3	4
Q354	Safe delivery	1	2	3	4
Q355	Antenatal care	1	2	3	4
Q356	Postnatal care	1	2	3	4
Q357	Safe abortion	1	2	3	4
Q358	HIV testing and counseling	1	2	3	4
Q359	Antiretroviral therapy	1	2	3	4
Q360	Prevention of mother to child transmission of HIV	1	2	3	4
Q361	Support for gender-based violence	1	2	3	4

Coding for missing codes is as follows (do not read them and code only if the respondent mentions them him-/herself):
-1 Don't know -3 Not applicable (filter)
-2 No answer/refused -5 Missing; Not applicable for other reasons

OBSERVATIONS BY THE INTERVIEWER

E. Respondent's interest (*Code how interested the respondent was during the interview*):
1 Respondent was very interested.
2 Respondent was somewhat interested.
3 Respondent was not interested.

E1. Respondent's literacy
1 Literate
2 Illiterate

F. Interview privacy (*Code whether the interview took place in privacy or not*):
1 There were no other people around who could follow the interview.
2 There were other people around who could follow the interview.

G. Code size of the place (city, village, but not the whole state) where the interview was conducted:

1	Under 2,000	5	20 - 50,000
2	2,000 - 5,000	6	50 - 100,000
3	5 - 10,000	7	100 - 500,000
4	10 - 20,000	8	500,000 and more

H. Code settlement type where interview was conducted:
1. Capital city
2. Regional center – state capital
3. District center – local government head quarters
4. Another city, town (not a regional or district center)
5. Village, any rural settlement

Q290A. Code ethnic group by observation

Yoruba	1
Hausa	2
Igbo	3
Fulani	4
Tiv	5
Ibibio	6
Other Africans	7
Asians	8
European	9
Others **[Write in]**____________________	10
**WRITE IN COUNTRY OF ORIGIN**________________________	11

Q 290B LANGUAGE SPOKEN AT HOME

English	1
Pidgin English	2
Yoruba	3
Igbo	4
Hausa	5
Tiv	6
Urhobo	7
Isoko	8
Itsekiri	9
Others **[Specify]**	10

Coding for missing codes is as follows (do not read them and code only if the respondent mentions them him-/herself):
-1 Don't know -3 Not applicable (filter)
-2 No answer/refused -5 Missing; Not applicable for other reasons

K2. Time of the interview:

End hour		End minute	

L. Interviewer's number: ____________

O. GEOGRAPHICAL COORDINATES

	+ or -	X	X	.	X	X
**O1. LONGITUDE**				.		
**O2. LATITUDE**				.		

P. LANGUAGE OF THE INTERVIEW

English	1
Yoruba	2
Igbo	3
Hausa	4

[*NOTE: if relevant, use codes appropriate to your own society*]

Q. Weight variable (*Provide a 4-digit weight variable to correct your sample to reflect national distributions of key variables. If no weighting is necessary, simply code each case as "1." It is especially important to correct for education. For example, if your sample contains 10 percent more university-educated respondents as there are in the adult population, members of this group should be downweighted by 10 percent, giving them a weight of .90*).

Coding for missing codes is as follows (do not read them and code only if the respondent mentions them him-/herself):
-1 Don't know -3 Not applicable (filter)
-2 No answer/refused -5 Missing; Not applicable for other reasons
