# Supplementary material for: Gender norms and women’s empowerment as barriers to facility birth: A population-based cross-sectional study in 26 Nigerian states using the World Values Survey
Source: PLoS One. 2022 Aug 18;17(8):e0272708. doi: 10.1371/journal.pone.0272708 (PMC9387800; doi:10.1371/journal.pone.0272708)
Supplement: S2 Appendix — (DOCX) [file pone.0272708.s002.docx]

*Table A. Beliefs related to maternal health care services and science/technology in general in Nigeria, results stratified based on gender.*

| **Beliefs related to maternal health care services and science/technology** |  | **States with high proportions of facility birth** | | ***p*-value ^a^** | **States with low proportions of facility birth** | | ***p*-value ^a^** |
| --- | --- | --- | --- | --- | --- | --- | --- |
|  |  | **Men** | **Women** |  | **Men** | **Women** |  |
| It is safer for a woman to give birth at a clinic than at home | N (%) | 316 (96%) | 306 (96%) | 0.93 | 173 (94%) | 164 (94%) | 0.94 |
|  | Mean value  1-4^b^ (SD) | 1.41 (0.60) | 1.40 (0.63) | 0.85 | 1.41 (0.69) | 1.48 (0.67) | 0.30 |
| I have confidence in hospitals | N (%) | 227 (69%) | 224 (70%) | 0.74 | 158 (85%) | 150 (85%) | 0.93 |
|  | Mean value  1-4^c^ (SD) | 2.06 (0.96) | 2.06 (0.91) | 0.99 | 1.58 (0.76) | 1.57 (0.82) | 0.83 |
| I have confidence in doctors | N (%) | 293 (91%) | 297 (94%) | 0.12 | 173 (94%) | 163 (95%) | 0.76 |
|  | Mean value  1-4^c^ (SD) | 1.54 (0.72) | 1.45 (0.65) | 0.09 | 1.37 (0.61) | 1.37 (0.60) | 0.96 |
| I have confidence in midwives at the clinic | N (%) | 244 (76%) | 257 (82%) | 0.07 | 160 (87%) | 145 (85%) | 0.56 |
|  | Mean value  1-4^c^ (SD) | 1.96 (0.87) | 1.83 (0.82) | 0.05 | 1.61 (0.79) | 1.57 (0.80) | 0.63 |
| I have confidence that health care facilities can provide safe delivery services | N (%) | 285 (89%) | 289 (92%) | 0.37 | 167 (91%) | 166 (95%) | 0.12 |
|  | Mean value  1-4^c^ (SD) | 1.50 (0.98) | 1.48 (0.89) | 0.81 | 1.38 (0.81) | 1.37 (0.64) | 0.82 |
| I have confidence in antenatal care | N (%) | 284 (91%) | 290 (91%) | 0.96 | 170 (93%) | 165 (95%) | 0.45 |
|  | Mean value  1-4^c^ (SD) | 1.36 (1.07) | 1.45 (0.75) | 0.18 | 1.33 (0.78) | 1.33 (0.62) | 0.98 |
| I have confidence in traditional birth attendants | N (%) | 122 (40%) | 118 (39%) | 0.79 | 117 (64%) | 100 (59%) | 0.29 |
|  | Mean value  1-4 ^d^ (SD) | 2.76 (0.96) | 2.77 (1.01) | 0.89 | 2.24 (1.00) | 2.32 (1.13) | 0.48 |
| Science and technology are making our lives healthier, easier, and more comfortable | N (%) | 295 (89%) | 274 (86%) | 0.21 | 147 (80%) | 142 (81%) | 0.69 |
|  | Mean value  1-10^d^ (SD) | 8.49 (2.24) | 8.14 (2.52) | 0.06 | 7.89 (2.59) | 7.75 (2.22) | 0.57 |
| Whenever science and religion conflict, religion is always right | N (%) | 279 (88%) | 280 (90%) | 0.37 | 174 (94%) | 168 (97%) | 0.27 |
|  | Mean value  1-4^b^ (SD) | 1.55 (0.89) | 1.56 (0.86) | 0.86 | 1.31 (0.61) | 1.29 (0.59) | 0.79 |
|  |  |  |  |  |  |  |  |
| **Total** |  | 331 | 322 |  | 185 | 175 |  |

^a^ Pearson chi-square for proportions and independent t-test for continuous variables

^b^ Strongly agree = 1, Strongly disagree = 4

^c^ A great deal of trust = 1, No trust at all = 4

^d^ Completely disagree = 1, Completely agree = 10

*Table B. Beliefs related to gender norms and empowerment in Nigeria, results stratified based on gender.*

| **Beliefs related to gender norms and empowerment** |  | **States with high proportions of facility birth** | | ***p*-value^a^** | **States with low proportions of facility birth** | | ***p*-value ^a^** |
| --- | --- | --- | --- | --- | --- | --- | --- |
|  |  | **Men** | **Women** |  | **Men** | **Women** |  |
| In the community where I live, men usually decide over health care visits and spending | Men decide N (%) | 133 (41%) | 98 (31%) | 0.007 | 116 (63%) | 98 (56%) | 0.20 |
|  | Mean value 1-9^b^ (SD) | 3.73 (2.28) | 4.44 (2.25) | <0.001 | 2.91 (2.07) | 3.17 (2.28) | 0.26 |
| In the community where I live, men usually decide if a woman should give birth at a clinic | Men decide N (%) | 99 (30%) | 91 (28%) | 0.61 | 100 (54%) | 100 (57%) | 0.56 |
|  | Mean value 1-9^b^ (SD) | 4.52 (2.38) | 4.63 (2.40) | 0.56 | 3.23 (2.24) | 3.18 (2.42) | 0.83 |
| In the community where I live, men usually decide over major household purchases | Men decide N (%) | 137 (42%) | 90 (28%) | <0.001 | 123 (67%) | 116 (66%) | 0.97 |
|  | Mean value 1-9^b^ (SD) | 4.02 (2.64) | 4.82 (2.58) | <0.001 | 2.77 (2.33) | 2.70 (2.25) | 0.77 |
| I experience freedom over my own life | N (%) | 243 (73%) | 227 (71%) | 0.52 | 115 (62%) | 87 (50%) | 0.02 |
|  | Mean value 1-10^c^ (SD) | 7.34 (2.68) | 7.12 (2.67) | 0.30 | 6.28 (2.99) | 5.72 (2.68) | 0.07 |
| Choice sub-index ^d^ | Mean value 0-1 ^d^ (SD) | 0.12 (0.17) | 0.11 (0.17) | 0.45 | 0.08 (0.13) | 0.08 (0.12) | 0.73 |
| Voice sub-index ^d^ | Mean value 0-1 ^d^ (SD) | 0.37 (0.26) | 0.38 (0.26) | 0.90 | 0.33 (0.25) | 0.34 (0.24) | 0.73 |
| Autonomy sub-index ^d^ | Mean value 0-1 ^d^ (SD) | 0.34 (0.32) | 0.31 (0.30) | 0.23 | 0.28 (0.29) | 0.23 (0.25) | 0.06 |
| Gender equality index ^d^ | Mean value 0-1 ^d^ (SD) | 0.34 (0.23) | 0.53 (0.26) | <0.001 | 0.21 (0.22) | 0.27 (0.26) | 0.009 |
|  |  |  |  |  |  |  |  |
| **Total** |  | 331 | 322 |  | 185 | 175 |  |

^a^ Pearson chi-square for proportions and independent t-test for continuous variables

^b^ Men decide=1, Both decide=5, Women decide=9

^c^ No choice at all = 1, A great deal of choice = 10

^d^ Higher value indicates more choice (coded by combining norms related to homosexuality, abortion, and divorce), voice (coded based on overall emancipative index), autonomy (coded by combining perceptions on independence, imagination, and nonobedience), and equality (coded by combining norms on women’s education, women’s employment, and women as politicians).

*Table C.* *Beliefs related to maternal health care services and science/technology in general, results only including states in northern Nigeria.*

| **Beliefs related to maternal health care services and science/technology** |  | **States in northern Nigeria with high proportions of facility birth** | **States in northern Nigeria with low proportions of facility birth** | ***p*-value ^a^** |
| --- | --- | --- | --- | --- |
| It is safer for a woman to give birth at a clinic than at home | N (%) | 122 (95%) | 327 (93%) | 0.45 |
|  | Mean value  1-4^b^ (SD) | 1.55 (0.61) | 1.45 (0.69) | 0.18 |
| I have confidence in hospitals | N (%) | 89 (70%) | 304 (87%) | <0.001 |
|  | Mean value  1-4^c^ (SD) | 2.05 (0.90) | 1.55 (0.77) | <0.001 |
| I have confidence in doctors | N (%) | 123 (97%) | 326 (94%) | 0.25 |
|  | Mean value  1-4^c^ (SD) | 1.50 (0.59) | 1.36 (0.61) | 0.03 |
| I have confidence in midwives at the clinic | N (%) | 96 (76%) | 297 (86%) | 0.02 |
|  | Mean value  1-4^c^ (SD) | 1.98 (0.85) | 1.59 (0.79) | <0.001 |
| I have confidence that health care facilities can provide safe delivery services | N (%) | 101 (80%) | 324 (93%) | <0.001 |
|  | Mean value  1-4^c^ (SD) | 1.89 (0.92) | 1.37 (0.73) | <0.001 |
| I have confidence in antenatal care | N (%) | 109 (87%) | 325 (94%) | 0.02 |
|  | Mean value  1-4^c^ (SD) | 1.66 (0.93) | 1.32 (0.71) | < 0.001 |
| I have confidence in traditional birth attendants | N (%) | 47 (39%) | 213 (62%) | < 0.001 |
|  | Mean value  1-4 ^d^ (SD) | 2.71 (1.05) | 2.27 (1.08) | < 0.001 |
| Science and technology are making our lives healthier, easier, and more comfortable | N (%) | 107 (84%) | 279 (80%) | 0.34 |
|  | Mean value  1-10^d^ (SD) | 8.42 (2.45) | 7.76 (2.42) | 0.009 |
| Whenever science and religion conflict, religion is always right | N (%) | 105 (85%) | 333 (95%) | < 0.001 |
|  | Mean value  1-4^b^ (SD) | 1.69 (0.89) | 1.30 (0.60) | < 0.001 |
| **Total** |  | 128 | 350 |  |

^a^ Pearson chi-square for proportions and independent t-test for continuous variables

^b^ Strongly agree = 1, Strongly disagree = 4

^c^ A great deal of trust = 1, No trust at all = 4

^d^ Completely disagree = 1, Completely agree = 10

*Table D.* *Beliefs related to gender norms and empowerment, results only including states in northern Nigeria.*

| **Beliefs related to gender norms and empowerment** |  | **States in northern Nigeria with high proportions of facility birth** | **States in northern Nigeria with low proportions of facility birth** | ***p*-value ^a^** |
| --- | --- | --- | --- | --- |
| In the community where I live, men usually decide over health care visits and spending | Men decide N (%) | 67 (52%) | 212 (61%) | 0.106 |
|  | Mean value 1-9^b^ (SD) | 3.58 (2.22) | 2.99 (2.16) | 0.01 |
| In the community where I live, men usually decide if a woman should give birth at a clinic | Men decide N (%) | 60 (47%) | 199 (57%) | 0.05 |
|  | Mean value 1-9^b^ (SD) | 4.10 (2.61) | 3.14 (2.36) | <0.001 |
| In the community where I live, men usually decide over major household purchases | Men decide N (%) | 67 (52%) | 238 (68%) | 0.002 |
|  | Mean value 1-9^b^ (SD) | 3.33 (2.17) | 2.66 (2.25) | 0.004 |
| I experience freedom over my own life | N (%) | 67 (52%) | 192 (55%) | 0.60 |
|  | Mean value 1-10^c^ (SD) | 5.93 (2.79) | 5.89 (2.82) | 0.89 |
| Choice sub-index ^d^ | Mean value 0-1 ^d^ (SD) | 0.13 (0.19) | 0.08 (0.12) | <0.001 |
| Voice sub-index ^d^ | Mean value 0-1 ^d^ (SD) | 0.26 (0.23) | 0.33 (0.25) | 0.003 |
| Autonomy sub-index ^d^ | Mean value 0-1 ^d^ (SD) | 0.38 (0.36) | 0.26 (0.27) | <0.001 |
| Gender equality index ^d^ | Mean value 0-1 ^d^ (SD) | 0.37 (0.23) | 0.23 (0.23) | <0.001 |
| **Total** |  | 128 | 350 |  |

Pearson chi-square for proportions and independent t-test for continuous variables

^b^ Men decide=1, Both decide=5, Women decide=9

^c^ No choice at all = 1, A great deal of choice = 10

^d^ Higher value indicates more choice (coded by combining norms related to homosexuality, abortion, and divorce), voice (coded based on overall emancipative index), autonomy (coded by combining perceptions on independence, imagination, and nonobedience), and equality (coded by combining norms on women’s education, women’s employment, and women as politicians).
